# Supplementary material for: Rewritable printing of ionic liquid nanofilm utilizing focused ion beam induced film wetting
Source: Nat Commun. 2024 Apr 5;15:2949. doi: 10.1038/s41467-024-47018-9 (PMC10997651; doi:10.1038/s41467-024-47018-9)
Supplement: Supplementary file 1 — Supplementary Information [file 41467_2024_47018_MOESM1_ESM.pdf]

Supplementary Information for

Rewritable printing of ionic liquid nanofilm utilizing focused ion beam induced film wetting

Authors: Haohao Gu[1]†, Kaixin Meng[1]†, Ruowei Yuan[1], Siyang Xiao[2], Yuying Shan[1], Rui Zhu[3], Yajun Deng[4], Xiaojin Luo[5], Ruijie Li[5], Lei Liu[5], Xu Chen[6], Yuping Shi[5], Xiaodong Wang[6], Chuanhua Duan[2], Hao Wang[1*]

Affiliations:

[1] Laboratory of Heat and Mass Transport at Micro-Nano Scale, College of Engineering, Peking University; Beijing, 100871, P. R. China.

[2] Department of Mechanical Engineering, Boston University; Boston, 02215, Massachusetts, USA.

[3] Electron Microscopy Lab, School of Physics, Peking University; Beijing, 100871, P. R. China.

[4] Future Technology School, Shenzhen Technology University; Shenzhen, 518118, P. R. China.

[5] School of Materials Science and Engineering, Peking University; Beijing 100871, P. R. China.

[6] Research Center of Engineering Thermophysics, North China Electric Power University; Beijing 102206, P. R. China.

*Corresponding author. Email: wanghpku@pku.edu.cn

†These authors contributed equally: Haohao Gu, Kaixin Meng.

The PDF file includes:

Supplementary Note 1 to 7
Supplementary Figures 1 to 22
Supplementary Tables 1 to 4
References 64 to 80

Supplementary Text

- **Supplementary Note 1: HFIB effects analysis**

HFIB effect on the solid substrates' morphology and chemical components

A PECVD SiO_2 substrate with a micro hole to calibrate the scan area of HFIB is employed for further tests of the HFIB possible effect on the solid surface's morphology characters and chemical components. A marked $5\ \mu m \times 5\ \mu m$ area is scanned with the following ion beam parameters: $I = 1\ pA$, $\tau = 2\ \mu s$, $s_i = 2\ nm$, where the subscript i stands for horizontal, h, and vertical, v. The substrate is then characterized employing an environmental scanning electron microscopy (ThermoFisher Quattro ESEM) for morphology change detection and with the results presented in the insets of Supplementary Fig. 16c. The SEM results indicate no significant nano scale bulging as reported by Chen et.al[1] which would induce significant changes in surface wettability. The relative low dose density and small mass of He ions lead to insignificant scatter effect as indicated by the Monte-Carlo simulation results.

Due to the larger beam spot of X-ray, a larger area $30\ \mu m \times 30\ \mu m$ was scanned by the HFIB with the following parameters: $I = 1\ pA$, $\tau = 2\ \mu s$, $s_i = 2\ nm$. To eliminate the environmental effect, the PECVD SiO_2 substrate has been annealed and the vacuum chamber of HIM has been cleaned by Plasma for $4\ h$ to diminish the possible adsorption of functional groups. An X-ray photoelectron spectroscopy (hermo Scientific™ Nexsa™) is then employed to detect the chemical components of the pristine and the irradiated substrate. As can be seen in Supplementary Fig.9c, the characteristic peaks remain identical before and after the irradiation.

The surface characterization result of current section indicates that in our IBFW experiments, the HFIB cannot manipulate the surface wettability and consequently to induce IL directional flow as discussed by previous researchers[1–4].

HFIB dose required to manipulate the surface wettability

In this section, we discuss the ion beam parameters that are sufficient to induce significant change of the PECVD SiO_2 substrate's wettability, which is much larger than the dose range adopted in IBFW experiments. The results further manifest rather different features in the ESEM figures of the solid substrate. The structure and the fabrication procedures of the microfluidic substrate can be found in literature [5].

The same PECVD SiO_2 substrate with a micro hole for spatial calibration is employed (the hollow circle in Supplementary Fig. 16a is the micro hole that connects the liquid reservoir beneath the surface and the surface of SiO_2) for the comparative experiment. The HFIB with different dose density is employed to irradiate a $3\ \mu m \times 3\ \mu m$ area (the red square in Supplementary Fig.16a,c), the irradiation dose increases from $5.6\ pC/\mu m^2$ to $100.7\ pC/\mu m^2$. Then [EMIM][DCA] is transferred into the liquid reservoir fabricated into the silica substrate. The IL would flow along the channel and form a liquid-vacuum interface at the micro hole (the bright circle in Supplementary Fig.16b, d, the IL is more conductive compared with the insulate substrate and the contrast can help the identification of liquid interface). If the solid substrate's wettability has not been modified, IL would not flow out from the micro hole due to the gravity and capillary force. On the other hand, if the solid surface has been modified by HFIB irradiation and become more hydrophilic a wetting film would form at the irradiated area.

Supplementary Fig.16a, c show the HIM figure of the micro hole and the irradiated square area, while panel A has been irradiated with $16.8\ pC/\mu m^2$ and figure C with $84\ pC/\mu m^2$. The

irradiated square is slightly brighter than the other area of silica surface due to the injected charges escaping from the insulate substrate and give rise to a different contrast to the imaging system of HIM. Supplementary Fig.16b, d are the HIM figures of the aforementioned two substrates with IL stored in their liquid reservoirs. In d, a wetting film of IL is formed in the square area which indicates a hydrophilic area modified by the high dose density ion injection, and the irregular bright and dark dots on the liquid film is the result of nano bulging at the silica substrate due to the sputter and atomic displacement effects. However, in b, the substrate contrast is identical with a, the much brighter contrast in the mciro hole (the white circle) indicates that even though the IL has filled the liquid channel and has reached the hole, the solid surface wettability has not been modified and IL cannot overcome gravity and capillary pressure to form a liquid film on the surface.

Supplementary Fig.16e is the ESEM image of the same PECVD SiO_2 substrate surface irradiated by $84\ pC/\mu m^2$ HFIB, with significant roughness elements can be identified. The higher roughness can explain the enhancement of hydrophilicity of the surface after high dose irradiation. Since the pristine surface has a 60° contact angle with [EMIM][DCA], the surface roughness would enhance the hydrophilicity between them.

Only ion beam with dose density higher than $84\ pC/\mu m^2$ can modify the substrate permanently and induce wettability-gradient liquid propagation, while the IBFW experiment typically involves dose density at $0.3{\sim}5\ pC/\mu m^2$ which is one-to-two orders of magnitude lower than the wettability modifying dose. Such results can demonstrate that the IBFW phenomenon reported in current work is completely different from the previous works that involve wettability manipulated surfaces by either chemical or physical gradients.

HFIB effect on the temperature of IL and thermocapillary

As reported in literatures[6,7], thermocapillary or Marangoni effect has been employed to trigger directional flow of liquid flow at micro scale. When injected He ions encounter the IL or solid substrate atoms and go through inelastic collisions, the kinetic energy of He ions would be transferred to the ions of IL and atoms of substrate. Most of the transferred energy would turn into heat and the temperature of IL and solid would be elevated. To eliminate the possibility that thermocapillary may induce the directional flow, the energy loss of He ions during irradiation process is estimated by means of Monte Carlo simulation.

The opensource software, Stopping and Range of Ions in Matter (SRIM)[8] is employed in current work as has been widely accepted in modeling the trajectories and interactions between FIB and samples. The MC system consists of a [EMIM][DCA] film with thickness $20\ nm$ above an amorphous SiO_2 solid layer with thickness $500\ nm$, more details about the IL can be found in Supplementary Table 2.

The MC simulation results of 100000 He ions with $30\ kV$ accelerate voltage injected to the aforementioned target material are shown in Supplementary Fig. 9 and 17. The trajectory lines of the first 100 He ions are shown in Supplementary Fig.9a, and 500 ions in the inset with $30\ nm$ of solid depicted. As can be seen, most of the He ions penetrate the IL film, with a vertical stop range within 500 nm, the He ions go through the IL film with little change in irradiation angle and go straight into solid region. The stopping range data indicate most of the inject ions vertical stop range higher than 150 nm, while the ions stop in IL or near the IL-SiO_2 interface can be neglected.

The energy loss curves of the He ions are depicted in Supplementary Fig.17. The energy loss of He ions consists of nuclear stopping and ionization stopping power. The ions energy and stopping power as functions of the inject depth are shown in Supplementary Fig.17c, d. Within the IL film region, He ions move in straight lines and the energy loss can be approximated as uniform

distribution (Supplementary Fig.17b), which can be expressed as $\frac{dE}{dz} \sim -9.07\ eV/Å$, with $8.26\ eV/Å$ attributed to ionization loss and $0.81\ eV/Å$ attributed to nuclear loss.

Heat transfer model assumptions

1. Source term of HFIB

   We assume that all the energy loss is converted into heat, and the energy gradient $\frac{dE}{dz} = -9.07\ eV/Å$ keeps constant in IL film. The heat flux can be expressed as:

   $$q \sim \frac{dE}{dz}\frac{I}{e\pi r_b^2}, \qquad (1.1)$$

   where $e = 1.6 \times 10^{-19} C$, at beam current $I = 1\ pA$, beam spot radius $r_b = 0.25\ nm$, the heat flux $q \sim 4.61 \times 10^{-10}\ W/nm^3$.

2. Geometrical boundary

   The interaction region of He ions and IL is simplified to a cylindrical system with a radius identical to beam spot, $r_b = 0.25\ nm$, and height consistent with the IL thickness 20 nm.

3. Heat transfer simplification

   We only consider heat conduction in the liquid film, zero heat convection in vacuum, and neglect the heat radiation effect due to the low temperature in chamber. We also consider no temperature gradient in the vertical direction. And the problem is simplified to be a 1-dimension heat transfer with cylindrical geometry, with Fourier number:

   $$Fo = \frac{k}{\rho c}\frac{\tau}{r_\infty} = 0.05, \qquad (1.2)$$

   where the thermal conductivity of [EMIM][DCA] $k = 0.2021\ W/(m \cdot K)$, thermal capacity $c = 1832.9\ J/(kg \cdot K)$, the dwell time of ion beam $\tau = 2\ \mu s$. The r_∞ can be estimated as $2\ \mu m$, therefore the bulk temperature of IL is $T = 20\ °C$ at $r \geq 2\ \mu m$.

Heat transfer governing equations and boundary conditions

The simplified governing equations at equilibrium are as follow:

$$\begin{cases} \frac{1}{r}\frac{\partial}{\partial r}\left(kr\frac{\partial T}{\partial r}\right) + \dot{q} = 0 & 0 < r \leq r_b \\ \frac{1}{r}\frac{\partial}{\partial r}\left(kr\frac{\partial T}{\partial r}\right) = 0 & r_b < r \leq r_\infty \end{cases}. \qquad (1.3)$$

Subjected to the following boundary conditions. Net heat flux is 0 at the beam boundary gives:

$$-k\frac{\partial T}{\partial r} = \frac{\dot{q}\pi r_b^2}{2\pi r_b} \qquad r = r_b. \qquad (1.4)$$

And bulk temperature at infinite boundary:

$$T_\infty = 20\ °C \qquad r = r_\infty. \qquad (1.5)$$

The geometrical symmetry give rise to zero heat flux at $r = 0$:

$$-k\frac{\partial T}{\partial r} = 0 \qquad r = 0. \qquad (1.6)$$

Solving Eq. (1.3-6) leads to an approximate temperature distribution as a function of r:

$$T(r) = \begin{cases} \frac{\dot{q}r_b^2}{2k}\ln\left(\frac{r_\infty}{r}\right) + T_\infty & r_b < r < r_\infty \\ \frac{\dot{q}r_b^2}{2k}\ln\left(\frac{r_\infty}{r_b}\right) + T_\infty & r = r_b \\ \frac{\dot{q}(r_b^2 - r^2)}{4k} + T(r_b) & 0 \leq r < r_b \end{cases}. \qquad (1.7)$$

The temperature reaches maximum at $r = 0$, $\Delta T_{max} = T(0) - T_\infty = 0.677\ K$.

Temperature gradient induced capillary force and effect on directional flow

It is well known that the surface tension as a function of temperature can induce directional flow of fluid. The temperature dependency of [EMIM][DCA] surface tension has been systematic studied. The surface tension decreases with temperature increasing[9] as $\frac{d\gamma}{dT} = -7.87 \times 10^{-5}\, N/(m \cdot K)$. With temperature gradient calculated from the last section, we get an estimation for the heating induced shear stress component:

$$\tau = \frac{d\gamma}{dT} \cdot \frac{dT}{dr} = 26.64\, Pa = 2.33 \times 10^{-9}\, dyn/cm^2. \qquad (1.8)$$

Kataoka *et.al* (*22*) reported a temperature gradient induced silicon oil flows experiment with the shear stress $\tau_{oil} = 0.73 - 0.80\, dyn/cm^2$, which is of 8 orders of magnitude larger than the HFIB heating effect. Additionally, the disjoining pressure induced pressure jump estimated in Supplementary Note 4 is of 2 to 3 orders of magnitude larger. As a result, we conclude that the heating effect of HFIB irradiation is not the dominant factor in the triggering of IL directional flow and is quantitatively insufficient compared with experimental data from literature.

- **Supplementary Note 2: Charging and time analysis of IBFW involving procedures**

We conclude that the IBFW mechanism composes of 3 stages and falls into two different physical categories. The first and the secondary ion emission are electrostatic phenomena that involve ion-beam-sample interactions and surface charging dissipation processes. The disjoining pressure induced liquid flow, on the other hand, belongs to the hydrodynamics discipline.

Our previous discussion of the IBFW with independent steps is based on the assumption that the time scale of ion emission process is several orders of magnitude lower than the hydrodynamics process, which enables us to consider these processes independently and come to our conclusion that the IBFW hypothesis is quantitively acceptable. Here, we are going to make a thorough discussion about the electrostatic processes to get a time scale estimation and compare with the characteristic time scale of hydrodynamic process in Supplementary Note 4.

Surface charging analysis

The surface charging process of dielectric material (SiO_2 for example) under the irradiation of focus ion beams (Ga^+ or He^+) was thoroughly discussed in literatures, and according to Yogev *et.al.*[10] the charging accumulation and dissipation is manipulated by the following factors: (1) generation of electron-hole pairs in the solid by incident ions; (2) neutralization of the incident ions by the excited free electrons; (3) sputtering of the surface atoms; (4) charging due to the secondary ion-electron emission; (5) leakage of mobile electron-hole pairs to the silicon substrate; (6) induced shallow traps by the incident ions and a consequent preferred trapping relative to the deep traps.

Our discussion mainly follows the procedure proposed in literature. Except that, both shallow and deep traps captured charges matter in our calculation, due to the time scale in our experiment is very short (μs) instead of hours in Yogev's model. Another difference stems from the ion emission induced by surface charging effect, the IL ions emitted from the reservoir would electrostatically block the surface charges. The selection of FIB parameters are beam current $I = 1\, pA$, dwell time $\tau = 1\, \mu s$, beam step size $s = 0.5\, nm$, accelerate voltage $V = 30\, keV$. Here we just give the expression for the overall charging density as a function of time since the deduction details can be found in[10]:

$$\frac{dQ(t)}{dt} = P(1+\gamma_e) \cdot I(t) - \sigma \frac{Q(t)}{\epsilon_r \epsilon_0} - \frac{7}{4}\, Y\, I(t) \cdot \Omega_0 \frac{Q(t)}{R_p} - - \int_0^t J(t)dt. \qquad (2.1)$$

The RHS composes of 4 terms, which represent the electron-hole pairs accumulation induced by the ion incident and secondary emission, the leakage current of silicon dioxide, the sputtering yield induced charge reduction and the accumulation of emitted ions.

Ion incident components

The first term is the ion incident terms. Where P is the probability factor introduced to take the recombination of positive ions/holes with excited free electrons into account, which ranges from 0 to 1; γ_e is the secondary electron emission yield of SiO_2; $I(t)$ is beam current when $t < 1\ \mu s$, and $I(t) = 0$ when $t > 1\ \mu s$.

Leakage current components

The second term expresses the charging reduction attributed to the leakage current from the SiO_2 layer to the Si substrate. We simplify the leakage problem between the silicon dioxide layer and the silicon substrate into an infinitely large parallel plate capacitor, whose upper plate has a fixed charge density and the electrostatic potential at the lower plate is zero at infinity. Therefore, the leakage current can be simply estimated by the conductivity and the field strength.

Sputtering yield components

The third term expresses the charging reduction due to the sputtering process of the incident ions. Sputtering occurs when the incident ions transfer sufficient momentum to one or more target atoms, those located on or near enough to the interface would be ejected into the vacuum if enough kinetic energy is transferred from the FIB. Where, Y is the sputtering yield acquired from SRIM simulation, Ω_0 is the atomic volume which can be estimated by the average density of SiO_2, R_p is the ions stopping range from the simulation results of SRIM.

Emitted ion components

The last term is the accumulation of IL ions. Since the accumulation of the ions would electrostatically block the surface charges beneath, the contribution is expressed as the integral of ion emission rate, *J(t)*, over time.

According to the Eq. 2.1, we can obtain the overall relationship between the surface charge and time. Combining the Eq. 2 obtained in the main text about the relationship between the surface charge density and the electric field strength at the contact line of the IL droplet, we can calculate the relationship between the field strength and time. Finally, invoking the formula Eq. 3.1 about the field-induced ion emission, we obtained the curve of the concerning ion emission rate versus the scanning time. As shown in Fig. S9, we use a home-made Matlab code to obtain a tool that can facilely manipulate the FIB scanning parameters and material-related parameters to conduct the above calculations.

Ion emission analysis

Supplementary Fig 10 show the surface charge density ($ions/nm^2$), electric field (V/m) and emitted ions number (we use the characteristic emission radius and the time step 1 ns to get the ions number) over 5 μs with the FIB scan starting at 0 s and ceasing at 1 μs. The scan parameters are selected as beam current $I = 1\ pA$, dwell time $\tau = 1\ \mu s$,which is a typical configuration adopted in our IBFW experiment.

The surface charge density and the field strength curves have the same characteristics, see Supplementary Fig 10a. As the beam turned on, both quickly rise and reach the threshold that is sufficient to trigger significant ion emission; as the beam is turned off (1 μs), both gradually decay.

The ion emission number curve in Supplementary Fig 10b is similar to the impulse function, and an emission peak appears instantaneously when the charge accumulation and the field strength reach the critical value, and then quickly decays. This difference is attributed to the exponential

term related to the free energy change of the emission barrier in the field emission current density formula. As shown in the inset of Supplementary Fig 10b, its characteristics are completely consistent with the previous two under logarithmic coordinates.

Employing the aforementioned tool, we further explore the dependence of the ion emission rate on the ion beam parameters and material characteristics. It is found that the significant ion emission of the IL is very sensitive to the beam current variation, and very sensitive to the parameter variation that affects the charge accumulation *e.g.* the conductivity of the substrate. Supplementary Fig 10c shows the ion emission rate as a function of time on 7 substrates, and Supplementary Fig 10d illustrates the total ion emission number on different substrates. The influence of substrate conductivity over ion emission and IBFW film inducing is clear.

There are two reasons we did not include the flood gun current into calculation:

1. The flood gun can only work after the HFIB finishes an entire row (or entire frame) of scan spots. The NPVE scan spots array usually composes 1024×1024 scan spots, so the injection dosage of HFIB exceeds the flood gun three to six orders of magnitude.

2. The ion emission model considers HFIB scans a single spot or a very small area near the contact lien to induce ion emission, the flood gun most likely is not working under such conditions.

Only in a reasonably selected scanning parameter range and on an appropriate dielectric substrate can the ideal ion emission effect be achieved. Such results coincide with out experimental discovery that IBFW requires a dielectric substrate exposed to a carefully tuned HIM.

Time scale analysis

Following the discussion above, we estimate the time scales of the three-step process involved in the IBFW phenomenon.

1. Primary ion emission

First, after the dielectric surface is scanned by the ion beam with the parameters mentioned before for about 1 μs, the surface charge and the corresponding field strength reach the threshold. This triggers significant ion emission that accumulates $10^3 \sim 10^4$ ions on the charged surface area within a time scale of 10^0 ns. Then, as the dwell time is reached, the FIB stops injecting/moves to the next scan spot and the substrate charges decay rapidly due to the combined effects of electron-ion recombination, IL ion neutralization and leakage current, and drops below the threshold within a time scale of 10^1 ns. Therefore, the time scale of the initial ion emission is of 10^1 ns, which is 37 ns in Fig. S9.

2. Secondary ion emission

Since the number of the primary emitted ions is of the same order of magnitude as the surface charge, we simply assume that the time scale of the secondary ion emission is close to that of the primary emission, which is 10^1 ns.

Under experimental condition, however, the first two steps are deeply coupled. And in the case of strong surface charge, the initial charges may produce the emission of a large amount of mixed positive and negative ions or even microdroplets. We did not perform the corresponding MD simulation of such condition due to the high computational requirements, but similar results can be found in the related literatures.

3. Pressure gradient driven flow

Based on Note 4, we obtain the average flow velocity of the disjoining-pressure-driven liquid film as 10^1 $\mu s/m$, and the scan spot spacing in the experiment is of $0.5 \sim 1$ nm. Therefore, a reasonable time scale estimation for the flow process should be around $10^{-4} \sim 10^{-5}$ s.

Additional proof of surface charging effect

Apart from the experiment conducted in the main text Fig. 2, different method of adjusting HFIB dose can alter the IBFW effect. The injection dose, $D = I \times \tau$, can be regulated by controlling both beam current, I, and dwell time, τ. The critical maximum spacing between neighbor scan spots to induce liquid flow, s_c, is chosen to represent the electrostatic driving force that a single spot can provide. At given SCD, the electric field that is sufficient to induce IBFW, E^*, can only be achieved within a specific spatial range, which is termed as the maximum scan spacing s_c, beyond which the IBFW can no longer be triggered.

The relationship between s_c and D of each beam spot is plotted in Supplementary Fig 14b. Two symbols indicate different methods applied to regulate dose. The blue rhombuses represent increasing beam current from 1 pA to 7 pA at constant dwell time, $\tau = 100$ µs; while yellow circles represent the elongation of dwell time from 100 µs to 1000 µs at constant beam current, $I = 0.7$ pA. The maximum spot spacing, s_c, increases with incremental irradiation dose under both situations. Whereas, the enhancing effect of increasing I on s_c is more outstanding than increasing τ at the same injected dose level. These phenomena indicate that the kinetic balance of charging effect of HFIB on solid substrates accounts for the IBFW phenomenon. Over the entire irradiation process, the injection and dissipation of charges occur simultaneously, with longer dwell time and the same irradiation dose the dissipation increases rapidly, which explains the weaker dependency of s_c on τ. The linear fit of $s_c \sim D^{1/2}$ to data further corroborates our hypothesis that the electrostatic interactions play crucial role in flow inducing, due to its inverse-square nature is coincident with the experimental data.

In summary, the flow process in the third step is not only four orders of magnitude slower than the ion emission processes, but also one order of magnitude slower than the scan dwell time. Therefore, we come to the following conclusions:

- First, the flow process is much slower than the ion emission and ion beam scanning movement, so the idea of separating the IBFW into independent processes and modeling and verifying them in a separate manner in the main text is well justified.
- Second, the pressure difference driven flow process is the rate-determining step in the IBFW nanofluidic method. If appropriate fluid, dielectric substrate, etc. can be selected to reduce the flow resistance, the maximal flow length and the flow velocity could be further enhanced.

Supplementary Note 3: Electric filed driven ion emission calculation

From a mechanical perspective, the IL – vacuum surface is distorted by the electric field of surface charges and a bumping meniscus is formed. When the meniscus is distorted to be hemispherical, the vertical component of surface tension reaches maximum. Once the surface charge continues to increase, a significant ion emission would take place during which both ions, clusters and tiny droplets may emit from the interface[11,12].

At the tip of the meniscus, the interface reaches a mechanical balance between the electrostatic force and surface tension, which gives $\tau_n^e \sim 2\gamma/r^*$, where r^* is the characteristic curvature radius for significant ion emission. By invoking the Maxwell tensor law, the former can be expressed as $\tau_n^e = \epsilon_0/2\left[(\mathbf{E_n^v})^2 - \epsilon_r\left(\mathbf{E_n^l}\right)^2 + (\epsilon_r - 1)\mathbf{E_t^2}\right]$, where $\mathbf{E_n^v}$ and $\mathbf{E_n^l}$ are normal components of electric field on vacuum and liquid sides, $\mathbf{E_t}$ is the tangential component, , ϵ_0 is the vacuum permittivity, ϵ_r is the relative permittivity of the IL. Taking $\mathbf{E_n^l} = O\left(\mathbf{E}^*/\epsilon_r\right) \ll \mathbf{E}^*$ and $\mathbf{E_t} = 0$ at the tip by geometrical symmetry yields $\epsilon_0(\mathbf{E}^*)^2 \frac{\epsilon_r - 1}{2\epsilon_r} \sim 2\gamma/r^*$.

To get an estimation for the characteristic electric field for significant ion emission, we should consider the process from a microscopic point of view. The ion emission is often described as a kinetic process in which ions evaporate from liquid-vacuum interface. The emission current density reads:

$$j_e = \frac{k_B T}{h} \sigma \exp\left(-\frac{\Delta G - G(\mathbf{E_n^v})}{k_B T} \right), \quad (3.1)$$

where j_e is the current emitted per unit surface area, k_B is Boltzmann's constant, T is the liquid temperature, h is Planck's constant, σ is the local net charge density at the liquid-vacuum interface, ΔG is the Gibbs free energy barrier for an ion to be emitted, E_n^v is the local vacuum electric field normal to the interface. $G(\mathbf{E_n^v})$ is the reduction of solvation energy barrier due to the external electric field, assumed to take the form $G(\mathbf{E_n^v}) = \sqrt{\frac{q^3 \mathbf{E_n^v}}{4\pi\epsilon_0} \frac{\epsilon_r - 1}{\epsilon_r + 1}}$ by the Schottky hump, where q is the ion's charge. The solvation energy of emitted ion can be estimated by the Born model as $\Delta G = \left(\frac{27}{4}\pi\right)^{1/3} \frac{\gamma^{1/3} q^{4/3} (1-\epsilon_r)^{2/3}}{(4\pi\epsilon_0)^{2/3}}$, where γ is the liquid – vacuum surface tension. Noteworthily, the ion emission energy barrier can be altered significantly by the local curvature of the IL-vacuum surface[13], but we consider a simple 2-dimensional model in current work and exclude this factor. Such influence should be considered to explain the different performances of IBFW at the CTL of the same liquid reservoir in the future.

Since $\Delta G \gg k_B T$ in general situations, j_e can always be ignored until $\Delta G - G(\mathbf{E_n^v}) = O(k_B T)$, which gives

$$\mathbf{E_n^v} \sim \frac{4\pi\epsilon_0 (\Delta G)^2}{q^3} \equiv \mathbf{E}^*, \quad (3.2)$$

which is the critical field it takes to induce strong ion emission.

We assume that the surface charges uniformly distribute with constant charge density, σ_{surf}, across the entire region with width l_0, and the distance between the charge region center and the contact line is d, we can express the normal component of electric field in vacuum exerted by surface charges as:

$$\mathbf{E_n^v} \cong k_0 \frac{Q_{surf} l_0}{(d+r^*)(d+l_0+r^*)} cos^3 \alpha. \quad (3.3)$$

By plugging in the parameters of the RTIL employed in current experiment, Eq. 3.1-3 give us a rough estimation on the relationship between the charge density required to trigger IBFW and the distance between the starting position of HFIB scan (we assume that the scan spot coincides with the surface charging center) and the contact line of liquid reservoir, d:

$$Q_{\mathrm{surf}} \cong \lambda \frac{(d+r^*)^2 + l_0(d+r^*)}{k_0 cos^3 \alpha} \mathbf{E}^*, \quad (3.4)$$

where $r^* = \frac{q^6 \gamma}{4\pi^2 \epsilon_0^3 (\Delta G)^4} \sim 10^{-8} m$ and $\mathbf{E}^* \sim 10^{9\sim 11} V/m$ under the experimental conditions.

- **Supplementary Note 4: Pressure gradient driven flow model**

Model assumptions

1. The cross section of the front of the moving film keeps constant during the whole process, which is a rectangle area with constant width, d, and uniform film thickness, h.
2. The precursor film thickness consists of ion pairs induced by field emission process keeps constant, h_{min}, which guarantee a constant driving force during the propagation of liquid film.
3. The process is simplified to be a quasi-equilibrium situation, which indicate that the liquid flow speed u is only a function of vertical coordinate z and is independent of time t and horizontal coordinate x. The velocity of the head of the moving film consequently coincides with the average flow speed and the film shape is independent of x.
4. We adopt the slip length data $b \approx 10\ nm$ of ionic-liquid-SiO_2 interface from literature[14,15], and zero lateral shear stress is taken at the liquid vacuum interface. According to the literatures, the boundary slip length of IL-solid interface depends on the specific conditions[16], such as, the liquid solid combination, the surface roughness, the surface potential. In most cases, the slip length increases with the roughness. The boundary slip length at different surface potential has been reported to vary due to the structure of EDL adjust to different surface potential, ILs with higher conductivity can be influenced more easily[16]. A larger surface potential has been reported to have an absorbed ion lubrication layer which can further reduce the IL-solid interface friction[17], in our case this may further enhance the flow velocity. For a better understanding of the hydrodynamics behavior of IBFW flow, both AFM measurements and MD simulation can be conducted.

Modeling of flow velocity

The simplified 2-dimensional form of Navier-Stokes equation (4.1) is adopted and together with the zero-shear-stress condition (4.2) and the boundary slip condition (4.3) give rise to the flow velocity as a function of vertical coordination z (4.4).

$$\mu \frac{d^2\mathbf{u}}{dz^2} = \frac{d\mathbf{p}}{dx} \quad (4.1)$$

$$\frac{d\mathbf{u}}{dz}|_{z=h} = 0, \quad (4.2)$$

$$\mathbf{u}(0) = b \cdot \frac{d\mathbf{u}}{dz}|_{z=0}, \quad (4.3)$$

$$\mathbf{u}(z) = \frac{1}{2\mu}\frac{d\mathbf{p}}{dx} z^2 - \frac{h}{\mu}\frac{d\mathbf{p}}{dx} z - \frac{bh}{\mu}\frac{d\mathbf{p}}{dx}. \quad (4.4)$$

The flow rate can be calculated as

$$\mathbf{Q} = d \cdot \int_0^h \mathbf{u}(z)dz \sim -\frac{d \cdot h^2(h+3b)}{3\mu}\frac{d\mathbf{p}}{dx}. \quad (4.5)$$

With the average flow speed is expressed as

$$\mathbf{u_{ave}} = \mathbf{Q}/S \sim -\frac{h^2+3bh}{3\mu}\frac{d\mathbf{p}}{dx}. \quad (4.6)$$

According to the IBFW hypothesis and the MD results, the precursor film ahead of the propagation bulk film has a thickness comparable with the size of the ion pairs of the IL, in our case the [EMIM][DCA] pair namely. An appropriate estimation for h_{min} is taken as $6 \times 10^{-10}\ m$, and the excess disjoining pressure of the precursor film serves as the driving force with the interface curvature induced capillary pressure serving as the resisting force, which gives a rough estimation for the pressure gradient ($\mathbf{\Pi}(h) \ll \mathbf{\Pi}(h_{min})$ and is omitted)

$$\frac{d\mathbf{p}}{dx} \sim \frac{\Delta\mathbf{p}}{\Delta x} = \frac{\mathbf{\Pi}(h_{min})-\gamma\boldsymbol{\kappa}}{L} = \frac{\left[-\left(\frac{A_{sl}\cdot f-A_{ll}\cdot f'}{6\pi h_{min}^3}-s_p\exp\left(\frac{d_{min}-h_{min}}{l}\right)-8c_{IL}\cdot h_{min}^{-7}\right)-\gamma\boldsymbol{\kappa}\right]}{L} \quad (4.7)$$

Where s_p is the polar component of the spreading coefficient, d_{min} is the atomic cut-off distance, l is the correlation length, c_{IL} is the strength Born repulsion. The result from Note S5 is employed.

A rudimental estimation for the average flow speed *i.e.* the mesoscopic propagation speed of IBFW liquid film can be expressed as a function of the length of the flow pattern, the flow speed decreases monotonically with the increase of flow length:

$$\mathbf{U}\sim\mathbf{u_{ave}} = \frac{h^2+3bh}{3\mu}\cdot\frac{\mathbf{\Pi}(h_{min})-\gamma\boldsymbol{\kappa}}{L}. \quad (4.8)$$

The calculation result based on Eq. 4.8 is shown in Fig. 2h, represented by the blue line, with the experimental results represented by the orange stars. The decaying tendency of flow speed with the elongation of flow length agrees well. The discrepancy takes place at rather long flow length indicating the complex nature lies in the current situation that has not been captured by the crude model proposed. Despite the crudity of our model, the quantitative consistency during the initiating stage of film propagation suggest that the disjoining pressure gradient is a potential candidate account for the unique performance of IBFW phenomenon.

- **Supplementary Note 5: Film thickness calculation**

The fabricated liquid film at equilibrium should maintain pressure balance at the entrance of the film (the conjunction part between the liquid reservoir and liquid film). The disjoining pressure is the partial derivation of the excess Gibbs free energy of a thin film ionic system over film thickness, h, and can be expressed as follow,$\mathbf{\Pi}(h) = \mathbf{\Pi}_{VdW} + \mathbf{\Pi}_{acid} + \mathbf{\Pi}_{born}$, (5.1) where the $\mathbf{\Pi}_{VdW}$ is the Van der Waals component of disjoining pressure attributed by the apolar interaction, the $\mathbf{\Pi}_{acid}$ is the acid base component of disjoining pressure attributed by the polar interaction and the $\mathbf{\Pi}_{born}$ is the Born repulsion interaction which only dominates at extremely close distances.
The VdW term can be calculated through the following equation according to London-Hamaker theory ([18,19]),

$$\mathbf{\Pi}_{VdW}(h) = \frac{A_{sl}\cdot f-A_{ll}\cdot f'}{6\pi h^3}. \quad (5.2)$$

Where A_{sl} and A_{ll} are the Hamaker constants for the solid/liquid and the liquid/liquid interactions and f and f' are retardation functions. The required Hamaker constants can be calculated from the characteristic frequencies[20,21], ν_{ic}, and the limiting values of the dielectric constants of the liquid and the solid[22], ε_{i0}, with the subscript *i* representing liquid (l) or solid (s)

$$A_{sl} = \frac{27}{32}\,\frac{h\nu_{lc}\nu_{sc}}{\nu_{lc}+\nu_{sc}}\left(\frac{\varepsilon_{l0}-1}{\varepsilon_{l0+2}}\right)\left(\frac{\varepsilon_{s0}-1}{\varepsilon_{s0+2}}\right) \quad (5.3)$$

$$A_{ll} = \frac{27}{64}\,h\nu_{lc}\left(\frac{\varepsilon_{l0}-1}{\varepsilon_{l0+2}}\right)^2.$$

The retardation correction functions wherein are

$$h < \frac{3\lambda_c}{2\pi} \quad \begin{cases} f = 1.01 - 0.28p^{sl} + 0.0143(p^{sl})^3 - 0.00193(p^{sl})^4 \\ f' = 1.01 - 0.28p^{ll} + 0.0143(p^{ll})^3 - 0.00193(p^{ll})^4 \end{cases}. \quad (5.4)$$

Where $p^{il} = 2\pi h/\lambda_c^{il}$ with i representing liquid (l) or solid (s), λ_c^{il} are calculated from the arithmetic average of characteristic frequencies of the solid and the liquid.
With the dielectric and vibrational spectroscopy data from literatures of the ionic liquids adopted in the current experiments the characteristic frequencies and the limiting values of the dielectric constants are acquired and the disjoining pressure of IL film as a function of film thickness, h, is shown in Supplementary Fig 15d. The Van der Waals component of disjoining pressure dominates

over the entire range of film thicknesses. At equilibrium the capillary pressure at the entrance of liquid film and the disjoining pressure at the stable film thickness reach mechanical balance, as shown in Supplementary Fig 15a. The curvature of liquid-vacuum surface reaches maximum at the conjunction area of film and reservoir with an average value of $6 \pm 1\ \mu m^{-1}$, and the capillary pressure can be estimated as $\mathbf{P}_{Ca} = \gamma\, \boldsymbol{\kappa}$, where γ is the liquid-vacuum surface tension from literature and $\boldsymbol{\kappa}$ is the maxima interface curvature. As shown in Supplementary Fig 15, the stable film thickness predicted by the force balance between capillary force and disjoining pressure is around 30 to 40 nm.

According to the AFM results of liquid films fabricated by IBFW manifest highly uniform film thickness ranging from 20 to 40 nm, with most of them around 30 nm which shows great consistency with the prediction.

- **Supplementary Note 6: Molecular dynamics simulation system**

Force Field Selection

The selection of force fields and atomic interactions parameters play significant roles in the determination of simulation accuracy and reliability. The force fields and inter atomic parameters adopted in current work come from series papers of Padua and Lopes[23–26] which is based on the optimized potentials for liquid simulation (OPLS-AA) suggested by Jorgensen *et al.* (*62*) and has been widely used in ionic liquid modeling. The interaction energy is expressed as the function form in OPLS-AA :

$$V_{total} = \sum_{bonds} \frac{K_b}{2}(r - r_{eq})^2 + \sum_{angles} \frac{K_\theta}{2}(\theta - \theta_{eq})^2 + \sum_{dihedrals} \frac{K_\varphi}{2}[1 + \cos(n\varphi - \delta)] + \sum_{i<j} \left\{ 4\varepsilon_{ij}\left[\left(\frac{\sigma_{ij}}{r_{ij}}\right)^{12} - \left(\frac{\sigma_{ij}}{r_{ij}}\right)^{6}\right] + \frac{q_i q_j}{4\pi\varepsilon_0 r_{ij}} \right\}. \qquad (6.1)$$

Where the total potential energy is composed of bond stretch term (harmonic form), bond angle bend term (harmonic form), dihedral torsion term (OPLS form), Lennard-Jones potential term and Coulomb force term.

In purpose of verification our theory on the mechanism of the directional flow of ionic liquids on solid surfaces, the ion pair were chosen to be 1-ethyl-3-methylimidazolium (C2C1im+) and dicyanamide (dca-) as shown in Fig 6.1:

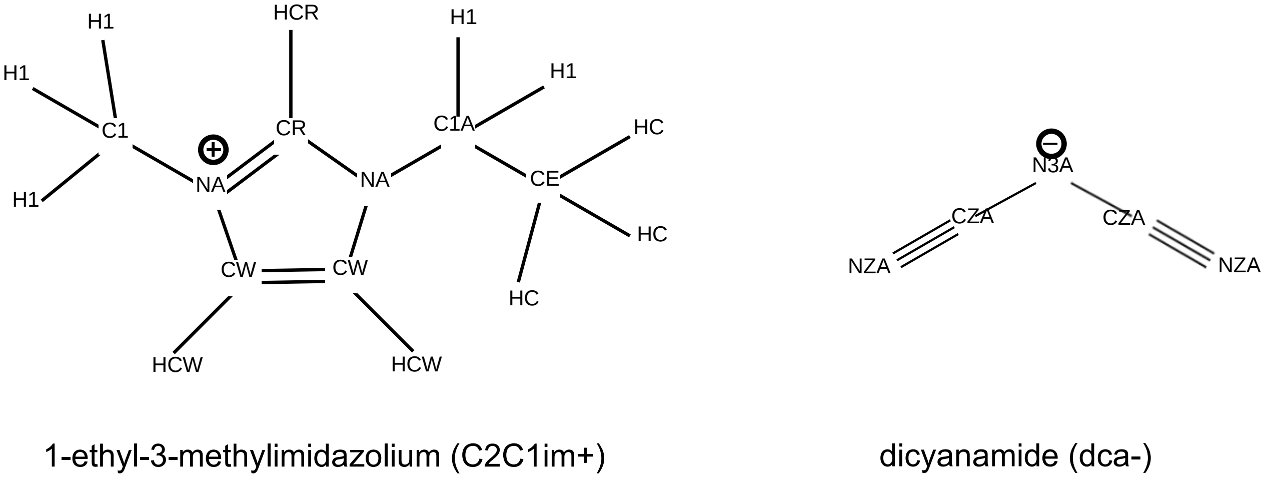

Figure 6.1: structural formula of C2C1imidazolium and dicyanamide.

The symbols in fig1 indicate that atoms of the same element were appointed to different parameters due to the distinct behaviors determined by local molecular structure, one of the advantages of adopting Padua's force field was their treatment to Nitrogen atoms make the simulation results can accurately reproduce experimental results of various species of imidazolium liquids. The parameters adopted in current work have been listed in Table 6.1.

Table 6.1 Ionic Liquid Force-Field Parameters (*58-62*)

atoms	m (g $\cdot mol^{-1}$)	q (e)	σ (Å)	ε (kJ $\cdot mol^{-1}$)
NA	14.007	0.15	3.25	0.71128
CR	12.011	-0.11	3.55	0.29288
CW	12.011	-0.13	3.55	0.29288
HCR	1.008	0.21	2.42	0.12552
HCW	1.008	0.21	2.42	0.12552
C1	12.011	-0.17	3.50	0.27614
H1	1.008	0.13	2.50	0.12552
C1A	12.011	-0.17	3.50	0.27614
CE	12.011	-0.05	3.50	0.27614
HC	1.008	0.06	2.50	0.12552
N3A	14.00	-0.76	3.25	0.71128
CZA	12.011	0.64	3.30	0.27614
NZA	14.001	-0.76	3.20	0.71128

bonds	r_{eq} (Å)	K_b $(kcal \cdot mol^{-1} \cdot Å^{-2})$[1]
CR - NA	1.315000	477.055449
CW - NA	1.378000	427.103250
CW - CW	1.341000	520.076482
C1 – NA	1.466000	336.998088
CR – HA	1.080000	339.985660
CW - HA	1.080000	339.985660
HC - CE	1.090000	339.985660
CE – C1	1.529000	267.925430
N3 - CZ	1.310000	502.629063
CZ - NZ	1.157000	650.000000

angles	θ_{eq} (°)	K_θ $(kcal \cdot mol^{-1} \cdot deg^{-2})$
CR – NA - CT	126.400000	70.004780
CW – NA – CR	108.000000	70.004780
CW – NA – CT	125.600000	70.004780
NA - CR – NA	109.800000	70.004780
NA - CR – HA	125.100000	35.002390

[1] The bond coefficients in LAMMPS soft package are slightly different from the others, the energy unit is kcal/mol and has a factor 2 because some write harmonic potentials as k/2 * (x - x0)^2 whereas others use K * (x - x0)^2.

NA - CW - CW	107.100000	70.004780
NA – CW - HA	122.000000	35.002390
CW – CW - HA	130.900000	35.002390
NA – CT - HC	110.700000	37.500000
HC – CT - HC	107.800000	32.994742
NA – CT - CT	112.700000	58.353250
CT – CT - HC	110.700000	37.500000
CZ – N3 - CZ	118.500000	43.260038
N3 – CZ - NZ	175.200000	50.788719

dihedrals	$K_1\ (kcal \cdot mol^{-1})$	$K_2\ (kcal \cdot mol^{-1})$	$K_3\ (kcal \cdot mol^{-1})$	$K_4\ (kcal \cdot mol^{-1})$
CT-NA-CR-NA	0	4.651052	0	0
CW-NA-CR-NA	0	4.651052	0	0
CT-NA-CR-HA	0	4.651052	0	0
CW-NA-CR-HA	0	4.651052	0	0
CR-NA-CW-CW	0	3.0	0	0
CT-NA-CW-CW	0	3.0	0	0
CR-NA-CW-HA	0	3.0	0	0
CT-NA-CW-HA	0	3.0	0	0
NA-CW-CW-HA	0	10.750478	0	0
HA-CW-CW-HA	0	10.750478	0	0
NA-CW-CW-NA	0	10.750478	0	0
CR-NA-CT-HC	0	0	0	0
CW-NA-CT-HC	0	0	0.124044	0
CR-NA-CT-CT	-1.259345	0	0	0
CW-NA-CT-CT	-1.709728	1.459465	0.189747	0
NA-CT-CT-HC	0	0	0.087715	0
HC-CT-CT-HC	0	0	0.3	0
NZ-CZ-N3-CZ	0	0	0	0
CR-CW-NA-CT	0	2.0	0	0
NA-NA-CR-HA	0	2.2	0	0
NA-CW-CW-HA	0	2.2	0	0

Construction and Initialization of Simulation Box

The ionic liquid coordinate and topology files of molecular dynamics simulation were generated by fftool python script with the assistance of packmol[27] package. The forcefield tool and force field data base provide the structure and force parameters of single ion pair, which served as input to packmol package to generate a file consists of 1200 ion pairs ($C_2C_1im^+$ and DCA^-) randomly distributed in a simulation box with appointed density. Then the LAMMPS data file can be created using fftool for a second time. In LAMMPS soft package, the uniform IL system go

through energy minimizing procedure for 10000 timesteps, then ran 50000 steps (50 ps) under NPT procedure with temperature and pressure fixed at 300 K and 1 bar respectively. The system volume recorded during the NPT simulation would be averaged and the system ran 100 ps NVT[28] ensemble simulation with averaged volume to get an initial IL droplet configuration for subsequent simulation.

The solid wall required for our system is SiO_2 glass to compare with the silicon wafer treated by thermal oxidation. Material Studio soft package was adopted, the example SiO_2 galss structure provided by MS was imported with supercell set to be (A = 20, B = 2, C = 4) to get enough space for ion emission and liquid film stretch in x direction. The solid system was constructed and annealed in Material Studio using the FORCITE module (after import and replicated the example structure, an energy minimization was carried out, then was heated to 2000K for 200ps and gradually cooled down to 300K), exported a data file which was consistent with LAMMPS format.

Once the solid and droplet data files were prepared, both systems were merged into one simulation box with the IL droplet set approximately 5Å above the SiO_2 surface. The size of the simulation box was set to be 43.8 nm, 90 nm, 11.2 nm in x, y, z directions respectively with periodic boundary condition on all three directions. The large spatial range in y direction is set to guarantee negligible electrostatic force in undesirable direction exerted by the surface charges, due to the PBC in all dimensions and the long-range nature of electrostatic force. The PBC in z direction guarantees the IL droplet to be an infinite film and eliminate the line tension influence on contact line region. To speed up the droplet spreading process on the surface, a small negative initial speed was appointed to all IL atoms once the simulation system was constructed, then the solid-fluid system ran 500 ps simulation with fixed NVT until the equilibrium state was reached (with no further decrease of the total energy and the contact angle approached experimental result 60-80°).

Surface charges injection and removal procedure

Once the simulation system achieve equilibrium, positive charges are randomly injected into the solid substrate, with positive ones distribute with 10 nm vertical and 5 nm width and 10 nm length. The charge is calibrated by a quadratic function of distance from the solid surface to a surface charge density. The calibrated surface charge densities range from $0.1 - 10\ e^{-} \cdot Å^{-2}$, and we choose $2.5\ e^{-} \cdot Å^{-2}$ in our subsequent simulation procedures. The temperature of the simulation system is adjusted from 100 K to 400 K with the Nose-Hoover thermostat[28], and the temperature effect on the surface charge density required to induce ion emission since the surface tension only alter slightly with increasing temperature, and 298 K is selected for the simulation procedure.

The injection of the surface charges is divided into 3 times (50%, 30%, 20%) for each injection-removing cycle, since the sudden increase in large number of charges within a small spatial range exerts drastic electrostatic forces on the IL ions and unphysical phenomena occur (the breaking of bonds and over-distortion of molecules). After each time of injection, the system is given 300 ps of relaxation time and approximately 1 ns total injection time for one cycle. Then the injected surface charges would be removed from the system and the system relaxes for 2 ns and give enough time for the formation of precursor film.

We depict the time averaged (0.5 ns) images of relative concentration (RC) of cations and anion, $ln\ (\frac{c_{cation}}{c_{anion}})$, where c_{ion} is the concentration of specific ions which is shown in Fig. S10C,D. By comparing the droplet profiles before and after four injection-removing cycles, a thin film of

ion pairs can be easily identified in Fig. S10d. The red and blue dots of the thin film is different from the droplet bulk character, which is dominated by neutral RC represented by yellow part. The neutral RC is the result of the time averaged thermal movement of ion pairs in bulk IL, while the red and blue dots adjacent to solid surface indicate that the ultra-thin ion pair film is restricted by the VdW interaction between solid and fluid.

The data processing and analysis are accomplished through the OVITO software[29] and homemade MATLAB codes.

Supplementary Note 7: Influence of radiolysis effect in IBFW

The ILs and the electrochemistry analytes in the main text remain intact through the IBFW experiments. The conclusion is base on the following discussion.

The imidazolium cations have shown good radiolytic stability. The dosage of HIB in our IBFW experiments is much smaller than the damage dosage reported in literature[30,31]. The radiolytic stability of imidazolium based ionic liquids under He^{2+} radiation were tested[1]. Decompose products H_2 were measured by NMR with irradiation dose ranges from 2 to 400 kGy, and no trace of reaction (<1%) was detected for 2 kGy irradiation. The irradiation dosage of Helium ion is 0.25 Gy during a typical IBFW experiment. For a $10\ \mu m \times 1\ \mu m$ film pattern with scanned area A fabricated from a 1 µL IL droplet with 1 pA beam current I, 1 µs dwell time τ, 1 nm scan spacing s, and 30 kV accelerate voltage U, the dosage can be calculated as:

$$D = \frac{Incident\ Energy}{Mass\ of\ IL} = \frac{U \cdot I \cdot \tau \cdot \frac{A}{s^2}}{V \cdot \rho} = \frac{30\times10^3 V \times \frac{10^{-12} C/s}{1.602\times10^{-19} C/e} \times \frac{10\times1 \mu m^2}{1\times1 nm^2}}{0.001 cm^3 \times 1.11 g/cm^3} = 0.25 \frac{J}{kg} = 0.25\ Gy.$$

The [$EMIM^+$] adopted in IBFW has a shorter alkyl chain attached to the aromatic ring, so the radiolytic stability of [$EMIM^+$] should be better than the [$BMIM^+$] tested in literature. Therefore, we believe that the cations of imidazolium are intact.

Anions structures also influence the radiolytic stability. The [DCA^-] is the most radiolytic stable anions among all anions[31]. The stability of a free radical decreases when the hybridization of the carbon goes from *sp*3 to *sp*2 to *sp*. Thus, it is hard to induce cleavage of the C≡N bond of [DCA^-] to form ·C=N.

Besides, the electrochemical results of the Ag(NTF_2) and TCNQ were consistent with literatures (main text Ref. [43, 44]), we believe they are not infected by the HFIB irradiation. In summary, the radiolysis effect on our sample can be negligible. As shown in Supplementary Fig. 9d, the FT-IR absorbance peaks of the liquid sample before and after the IBFW experiment are consistent, both indicate that the sample is [EMIM][DCA], no obvious radiolysis reaction takes place.

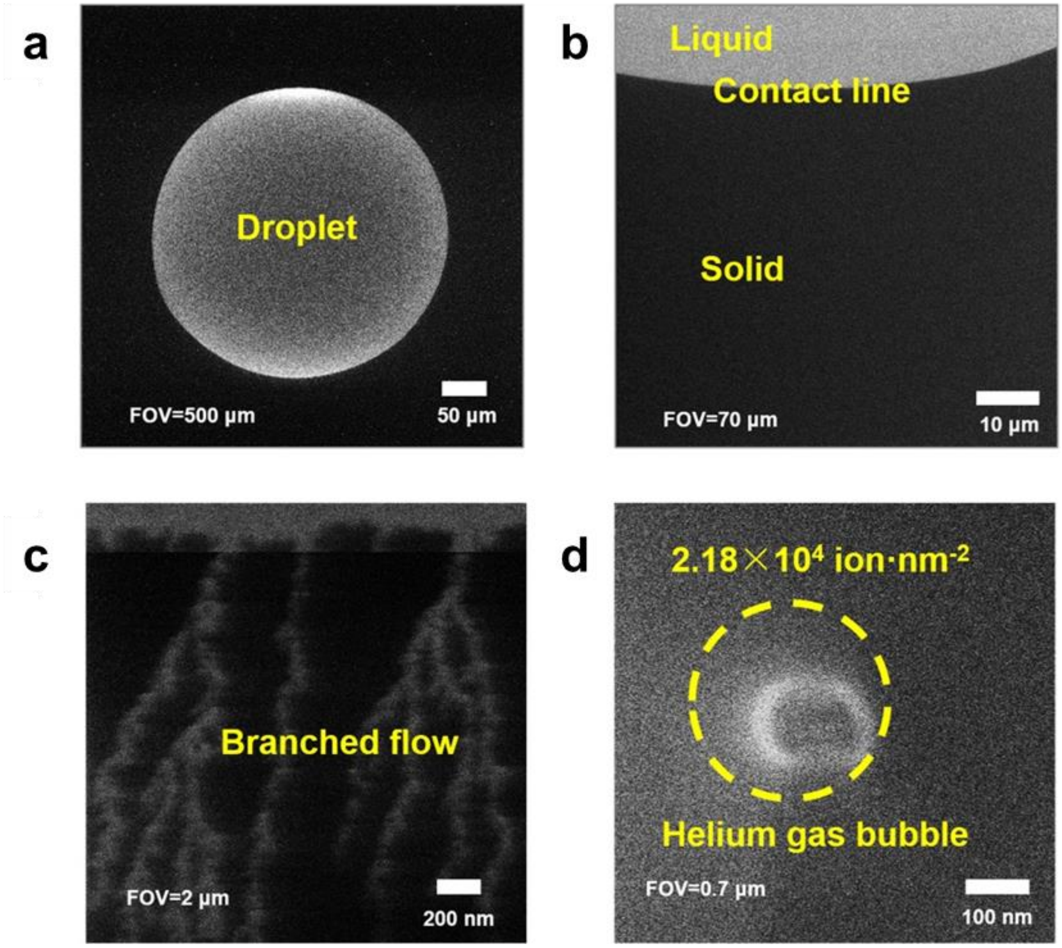

Supplementary Figure 1: HIM images of the examples of three working modes.

FOV means field of view, which is the length of the image. (**a**) The top view of an IL droplet on SiO_2 substrate under imaging mode. (**b**) Zoom in perspective of the contact line region of the droplet in (a). (**c**) The HIM image after a frame of scan under inducing mode. Branched flow character instead of uniformly propagation from contact line is captured. (**d**) The damaging mode of HIM. Helium gas bubble injected into IL under ultra-high dose density (2.184×10^4 ions/nm^2), both solid and liquid can be decomposed.

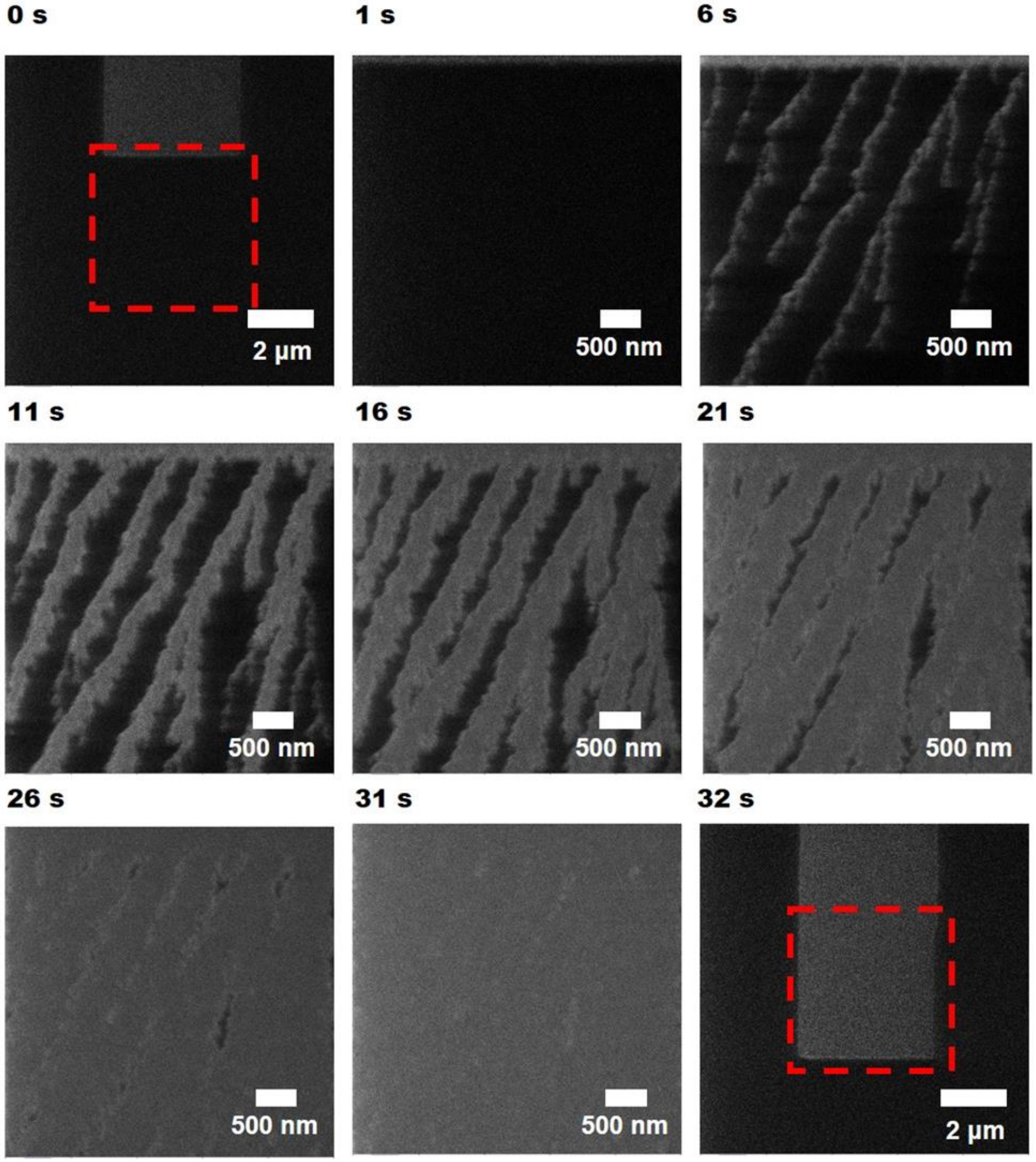

Supplementary Figure 2: The time series of HIM images of the propagating process of IBFW. IL flows over the contact line through several protrusions that located at the CTL and propagates separately as a series of long parallel 'rivulets' along the pattern direction (top-to-bottom). The rivulets branch off into secondary ones then multistage branching forms along the propagating direction. The exhibiting free-surface dendritic structures are completely distinct from commonly reported meniscus film fronts.

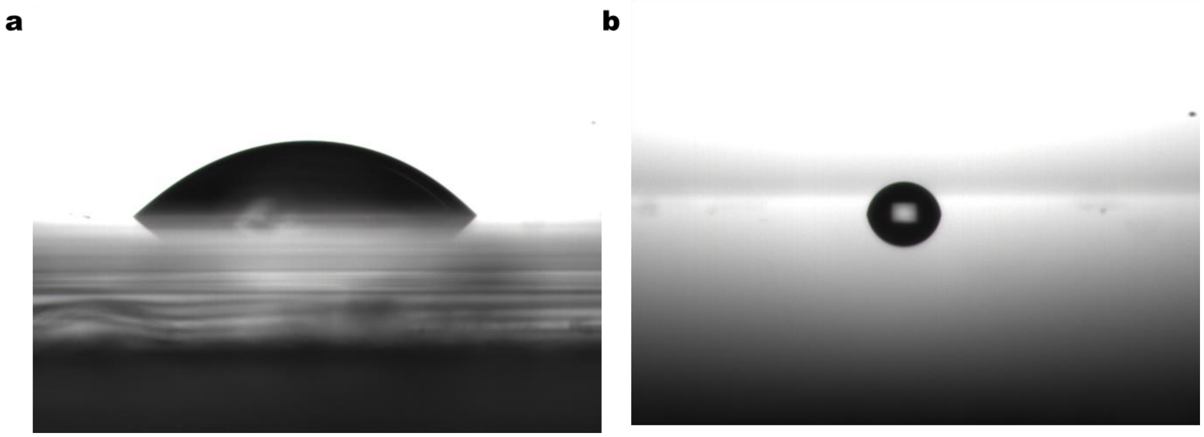

Supplementary Figure 3: Contact angle measurements of a droplet of [EMIM][DCA] on SiO_2 wafers.

(**a**) A droplet settled on a plasma-enhanced-chemical-vapor-deposition (PECVD) SiO_2 wafer. (**b**) A droplet settled on a thermal-oxidate (TOX) SiO_2 substrate. The contact angles are 59° and 76°, respectively.

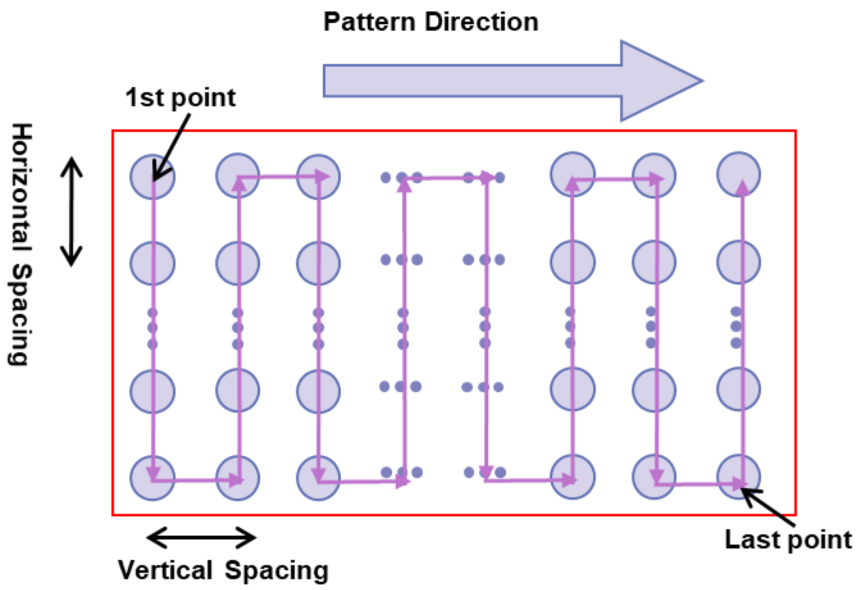

Supplementary Figure 4: The scan spot array adopted by NPVE software.

The large purple arrow illustrates the total scan/pattern direction. The NPVE automatically fills the designed pattern with a sequential of separate scan spots, and the HFIB with given parameters stop and scan a single spot for a designated time, namely the dwell time τ, before it moves to the next scan spot. The sequence of scan is determined by the NPVE and is represented by the thinner purple arrows, the vertical and horizontal spacing together determine the scan spot density, with no specific explanation both spacings are same throughout our experiments.

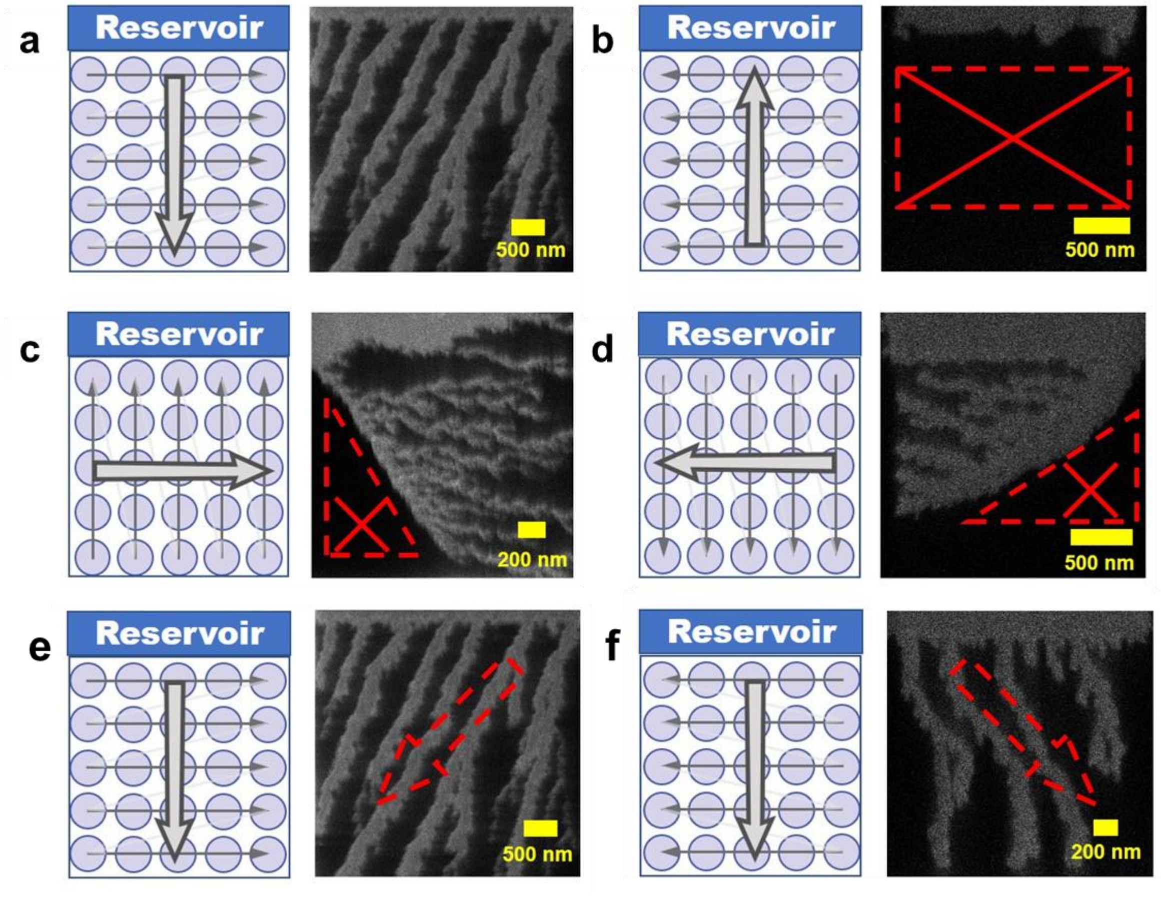

Supplementary Figure 5: The pattern direction effects on the IBFW inducing flow pattern.

(**a**) and (**b**) indicate that the scan direction should point outwards from the contact line of liquid reservoir while the opposite direction fails to induce liquid film flow. (**c**) and (**d**) show the result of the pattern direction parallel to the contact line with the spot-by-spot scan moving inwards and outwards from the contact line. Both scan methods fail to induce liquid film that well agrees the designed pattern. (**e**) and (**f**) serve as an illustration of the spot-by-spot scan direction can influence the tilting of liquid film while the final results are identical.

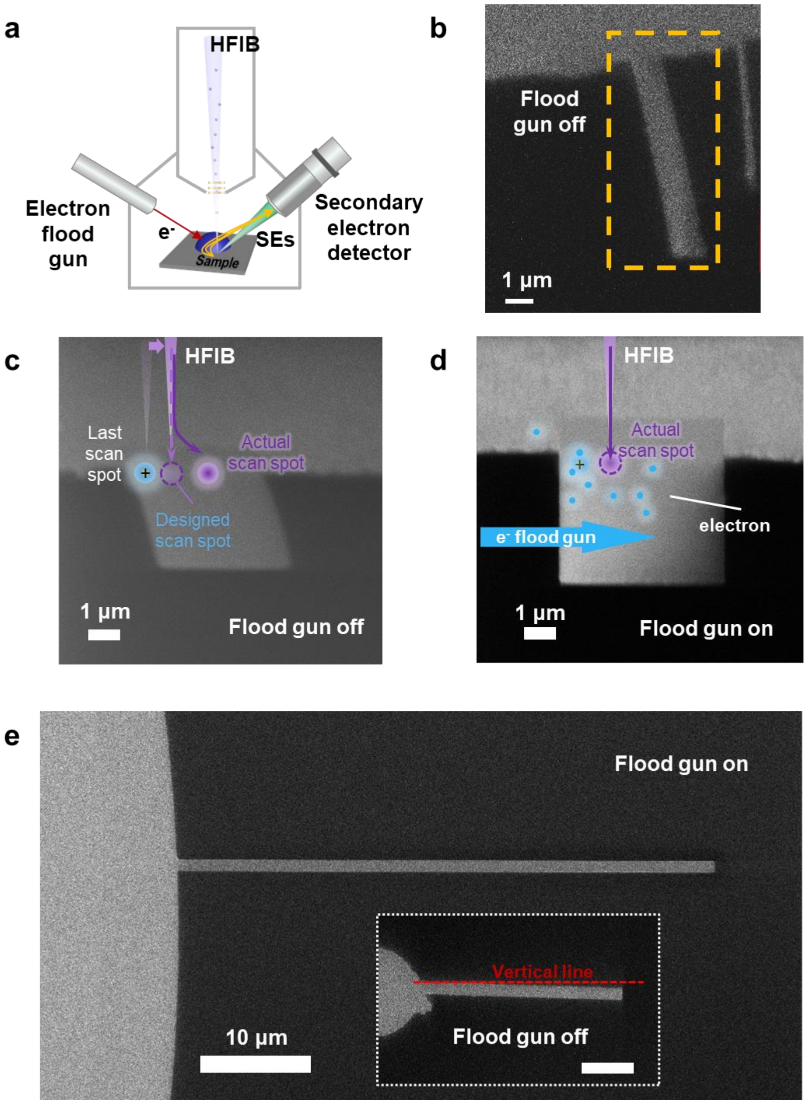

Supplementary Figure 6: The role played by flood gun (an instrument that can spray low energy electron flow to the sample during the HFIB scanning) in IBFW experiment. (a) Schematic diagram of the helium ion microscope. Helium focused ion beam scan vertically at the sample, the secondary electron detector can capture the electrons excited from the sample. The flood gun can spray electrons onto the HFIB scanned area while the ion beam and SE detectors are turned off. **(b)** The flood gun should be turned on or the film pattern will tilt. **(c)** If the dwell time becomes too long, the irradiated area carries large amount of positive charges and repels the ion beam when HFIB moves to the next spot. The HFIB will drift from design, consequently, the fabricated liquid film tilts toward the lateral scan direction (b, c). **(d)** By turning on the flood gun, the electrons spray on the solid surface, block the positive charges and the liquid films retain the designed patterns. **(e)** Moreover, the flood gun can only work after the HFIB finishes an entire row (or entire frame) of scan spots which makes the injection dosage of flood gun much smaller than HFIB. Consequently, the introducing of flood gun would not weaken the liquid inducing ability of HFIB (the scale bar of inset is 10 μm).

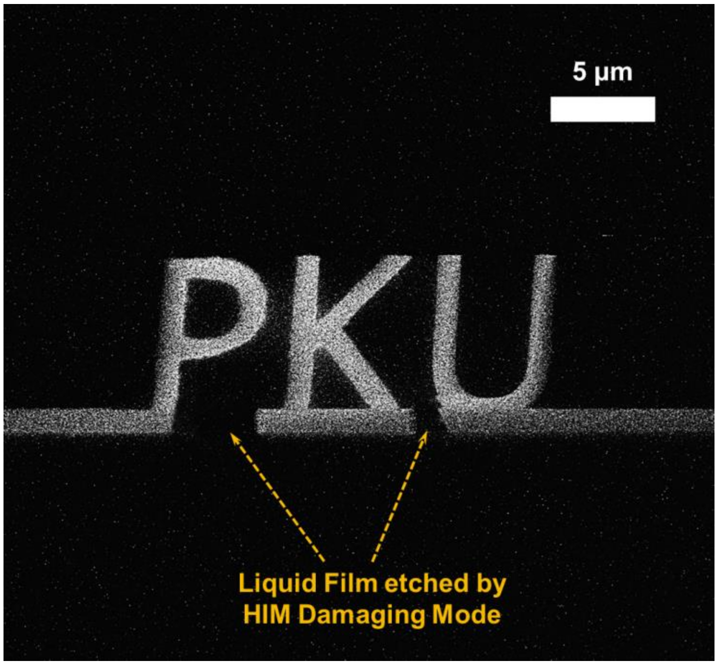

Supplementary Figure 7: Discontinuous pattern achieved by employing damaging mode of HFIB.

After the PKU letters pattern has been induced from a continuous liquid film channel, the conjunction parts between the film channel and the letters pattern are etched by HIM (by setting the scan dose density to 10^4 ions/nm^2, the liquid film is etched).

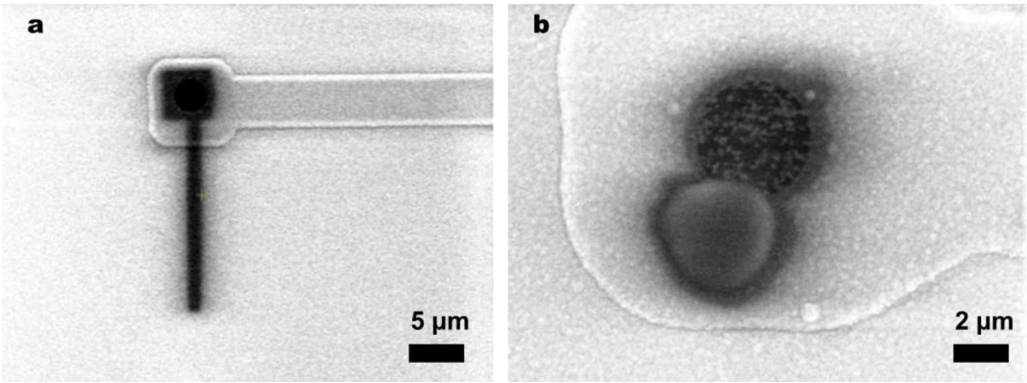

Supplementary Figure 8: SEM images of liquid film induced by ion and electron beams.
(a) 30 keV helium focused ion beam induced liquid film on a SiO2 substrate; **(b)** 5 keV electron beam induced liquid flow on the identical substrate. The liquid can also be induced to flow from the reservoir, but the flow pattern is out of control. The much more diffractive nature of the electron beam-sample interaction, and the consequent wider surface charges lateral distribution give rise to such results.

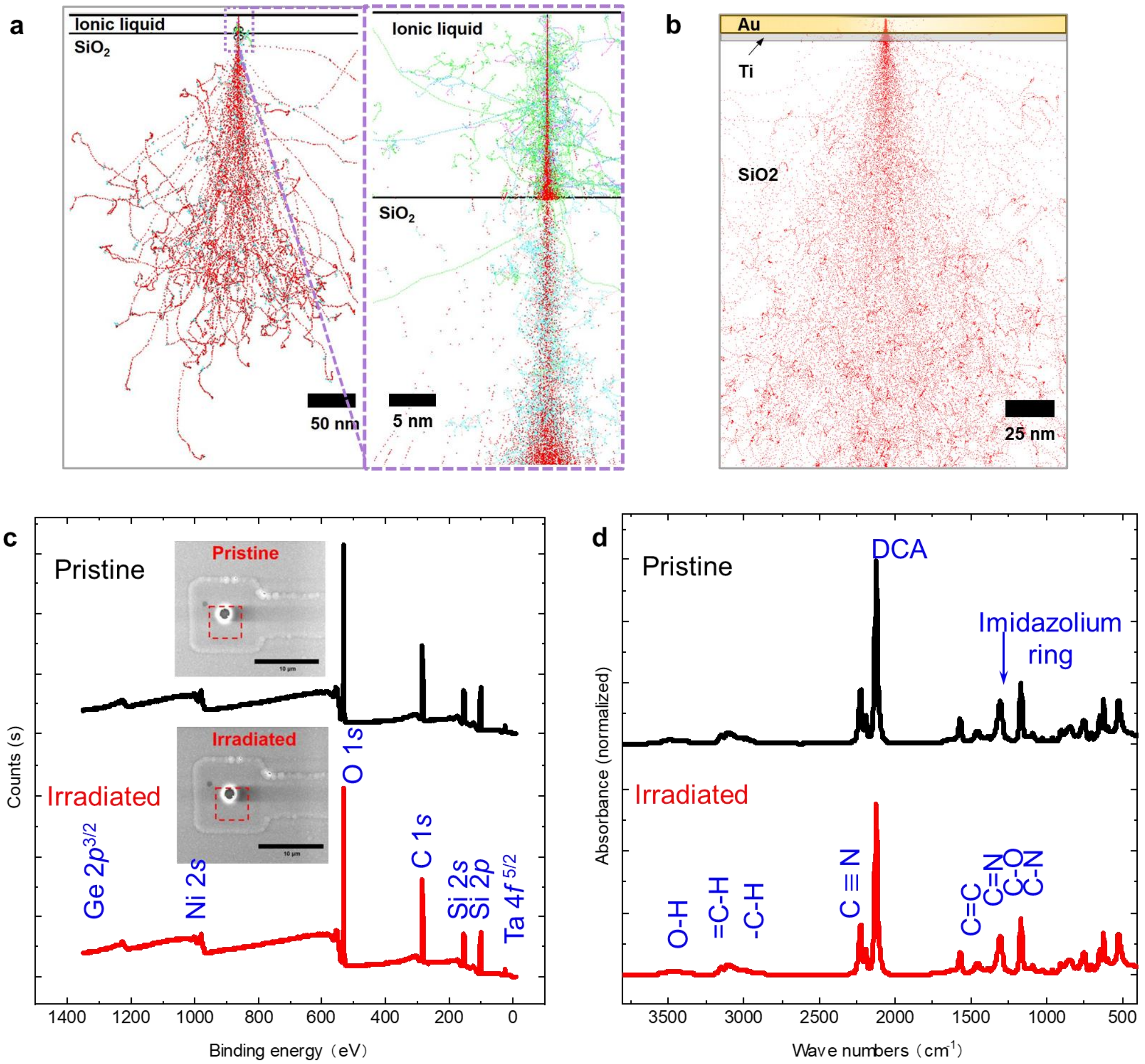

Supplementary Figure 9: The HFIB irradiation interaction with liquid and substrates under experimental conditions. (a) Monte Carlo simulation results of 3000 He ion irradiating a ionic liquid film of 30 nm thickness above silica substrate, the accelerate voltage is 30 kV to reproduce the experimental conditions. The right part is the magnification of He ions near liquid trajectory lines and spatial distribution. **(b)** the MC simulation results of He ions with 30 kV irradiate 10 nm Au, 5 nm Ti and 300 nm SiO_2. Similar to the results in the previous case, the HFIB can easily penetrate the first 15 nm layers of metal and deposit positive ions into the insulating SiO_2 substrate. Such results indicate that IBFW can also be done on insulating substrates with thin film of metal deposited on the surface. The vertical stopping range of He ions exceed 290 nm on both substrates. The lateral projection distances of He ions in both cases range from 90 nm to 110 nm in separated repeated simulations, which agree with the IBFW film resolution 100 nm. **(c)** the XPS spectra of the same substrate before (black) and after (red) the irradiation of HFIB under experimental conditions with insets show the ESEM results of pristine and irradiated area of solid surface. **(d)** the FT-IR results of ionic liquid before and after IBFW experiments confirm that the liquid sample go through no chemical reaction through the experiments. The absorbance peaks before and after

the IBFW experiment are consistent, both indicate that the sample is [EMIM][DCA], no obvious radiolysis reaction takes place.

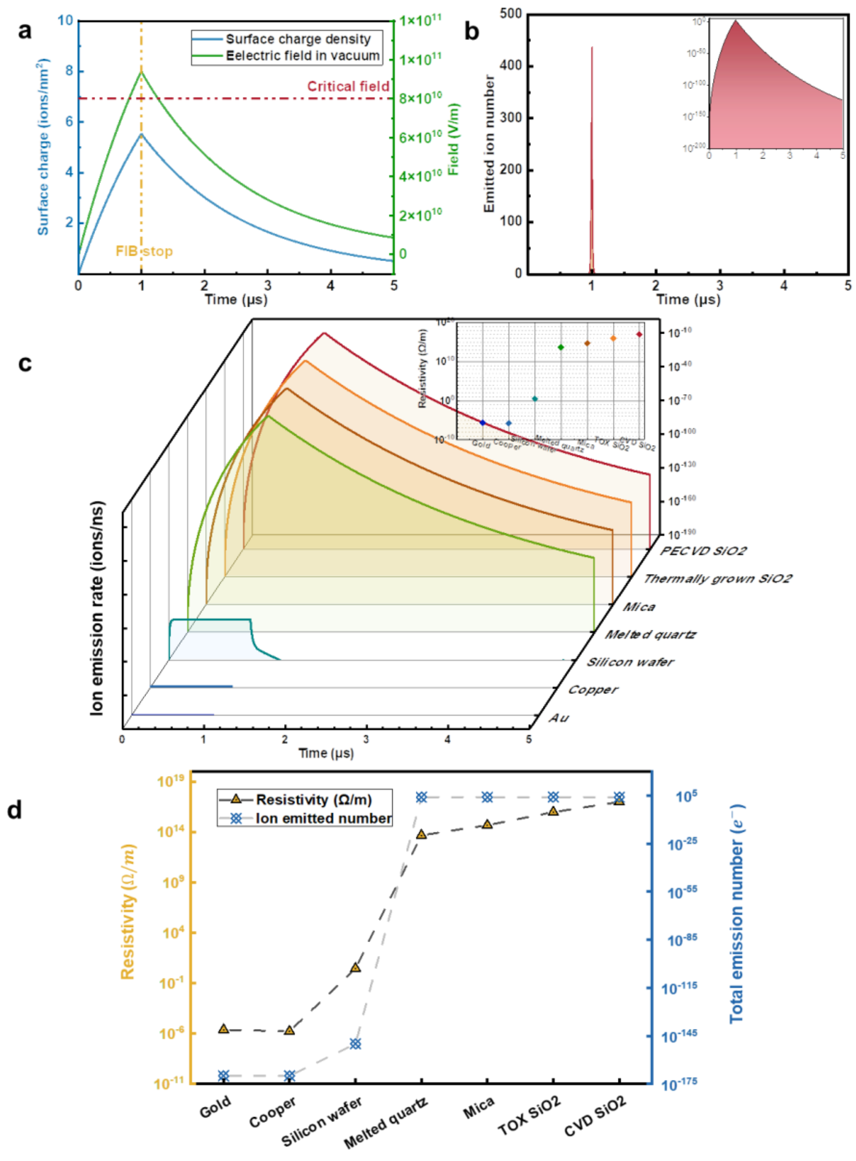

Supplementary Figure 10: The surface charging density, electric field in vacuum and consequent ion emission rate as a function of time.

(a) The HFIB beam current 1 pA, dwell time is 1μs, therefore from 0 μs to 1 μs the charge density and field strength increase rapidly then gradually decay. **(b)** The ion emission number only becomes significant when electric field exceeds the critical value, and only last for 37 ns, the inset shows the emission number under logarithmic coordinate. **(c)** The ion emission number as a function of time on different substrates. The dielectric substrates share similar emission characters while semiconductor substrate is different. On silicon, the conductivity far exceeds the dielectric ones, therefore the ion emission is very small and quickly reach equilibrium since the substrate charges' accumulation and dissipation reach a balance. (**d**) The total emission number over different substrates which is indicated by the blue squares, the resistivity of the substrates is also illustrated by yellow triangles for comparison.

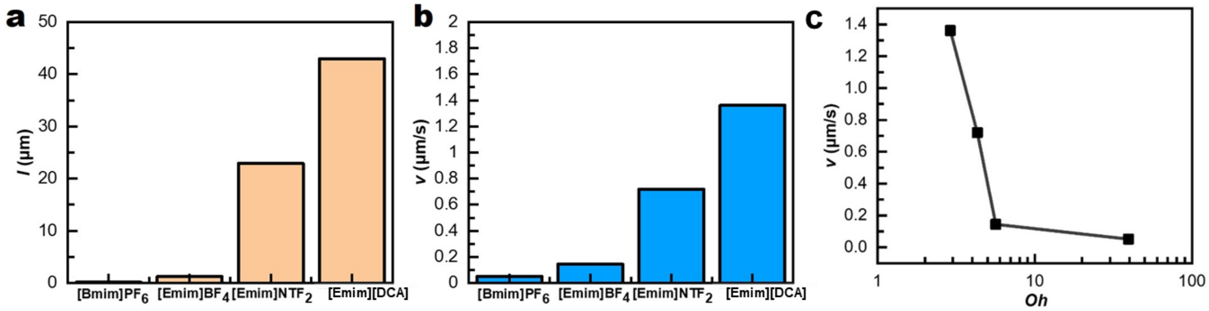

Supplementary Figure 11: the IBFW results of different ILs and the relationship with Oh number.

(a) Film lengths of different liquids on SiO_2 substrate can be induced with a same pattern. Scan parameters are 1 pA, 2.5 nm spacing, 3 µs dwell time, 50 × 1 µm pattern. **(b)** The flow velocity of different ILs. **(c)** The relationship between Ohnsorge number $Oh = \frac{\mu}{\sqrt{\rho d \gamma}}$ and flow velocity, the fluid data can be found in Supplementary Table 3.

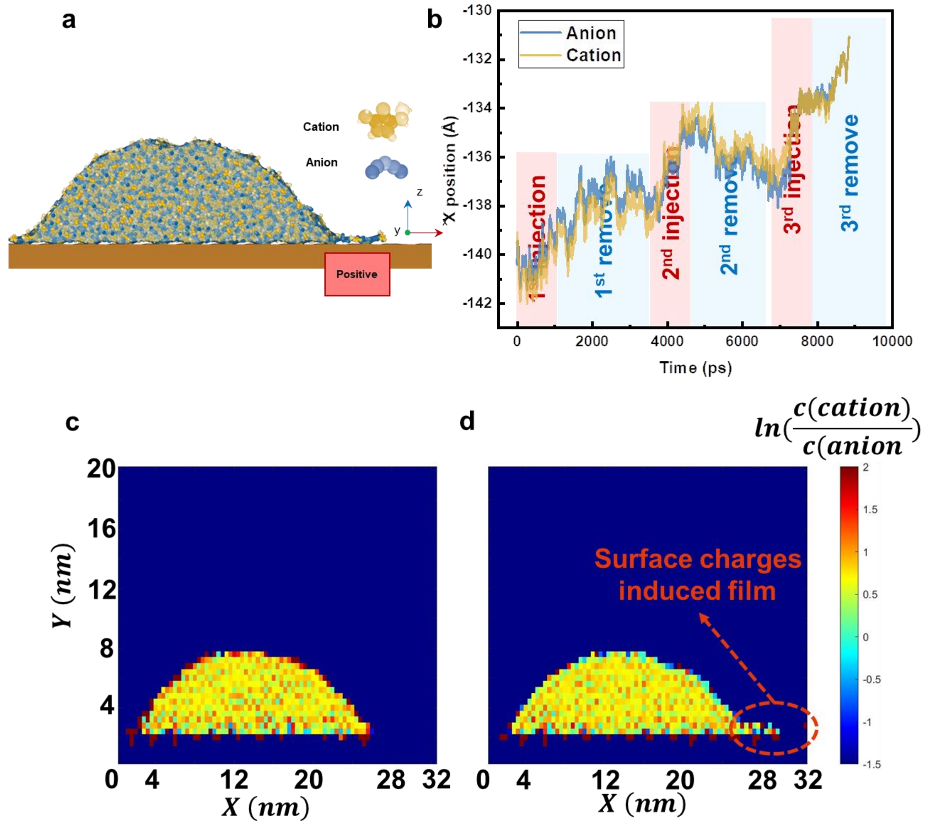

Supplementary Figure 12: Molecular dynamics simulation procedure and nanofilm induced by surface charge.

(**a**) Schematic of IL droplet settled on fused silica substrate with red rectangle indicates region in which positive charges are injected. (**b**) The X position of cations and anions mass center during the simulation as a function of time. Three steps of movement in the positive direction can be recognized in accordance with the surface charges injection circles for three times. (**c, d**) The relative concentration of cations and anions of the droplet after relaxation and the droplet after four cycles of surface charges injection-remove (4 nm moved to the right). The red dashed circle illustrate the precursor film induced by surface charges.

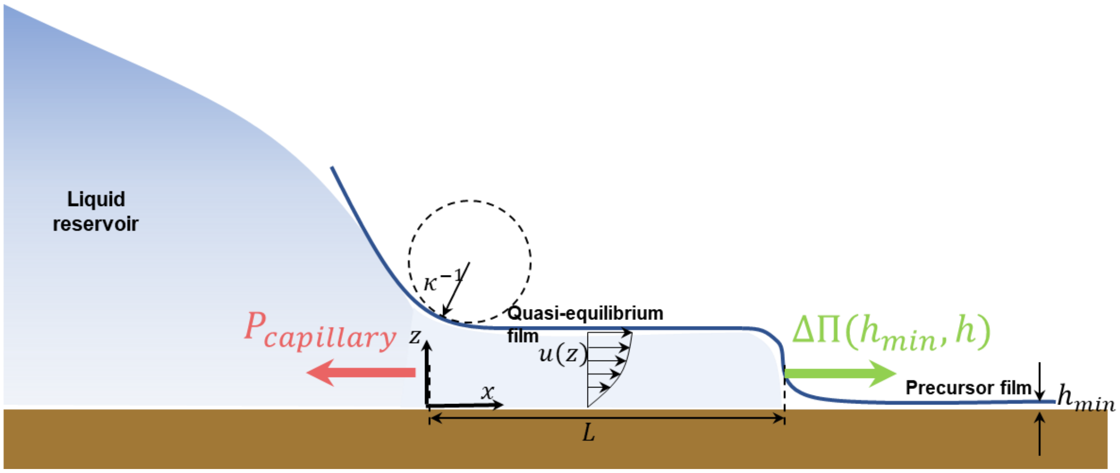

Supplementary Figure 13: Schematic for the propagating liquid film driven by the disjoining pressure.

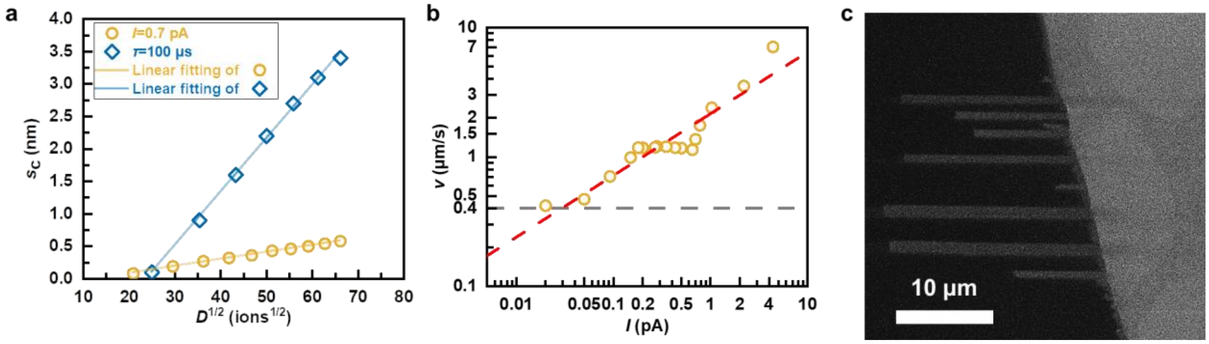

Supplementary Figure 14: Flow speed measurement experiments.

(a) The single spot scan style in HIM, which scans one-dimensionally along the pattern direction, is applied to analyze the interaction spatial range of a single irradiation spot quantitatively. The critical maximum spacing between neighbor scan spots to induce liquid flow, s_c, represents the upper limit for the interaction spatial range of the beam spot. When scan spot spacing exceed s_c, HFIB fails to induce continuous flow however large the dose is. The injection dose density, D, is regulated by changing beam current, I, at constant dwell time 100 μs, or changing dwell time, τ, at constant beam current 0.7 pA. The relationship between s_c and D of each beam spot is plotted. **(b)** Maximal flow velocity as a function of the beam current *I* for a 20 μm length rectangle pattern. The scan speed is changed by the pattern width. The flow speed increases with increasing beam current at same dwell time, 5 μs, and spacing 1 nm. **(c)** An example of the change width method for the measurements of flow velocity at different film length.

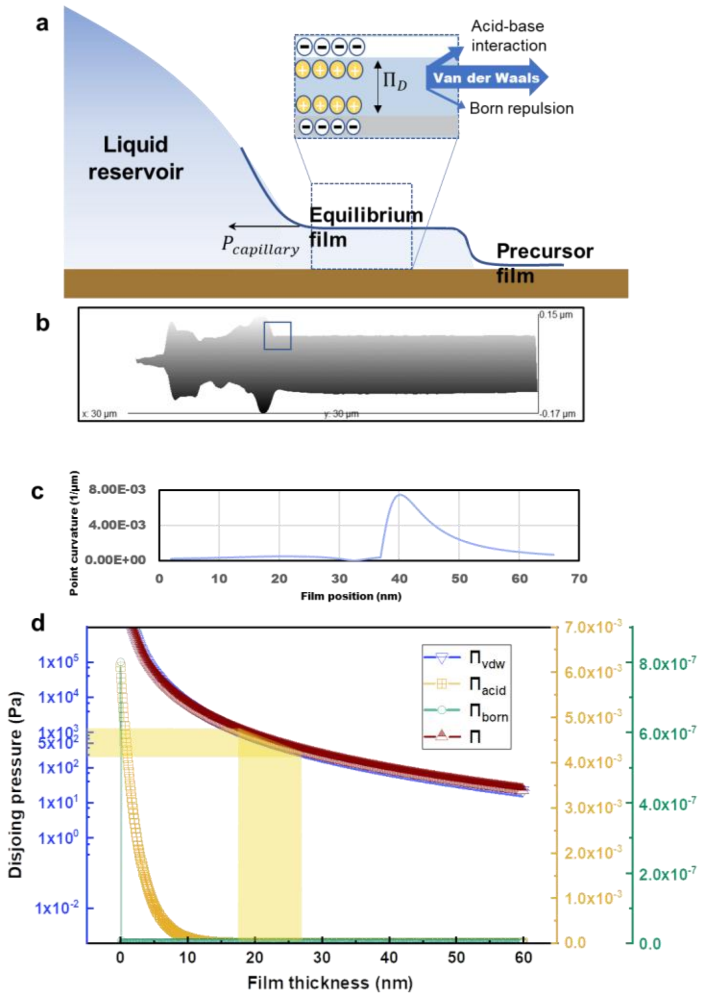

Supplementary Figure 15: The balance between the disjoining pressure and capillary force explains the film thickness range observed in experiments.

(**a**) The capillary force draws the liquid film at the entrance side tend to drainage the film while the overlapping of interfaces/ double-layers induces an excess pressure term, disjoining pressure, tend to thicken the film. The balance between them determines the stable film thickness. (**b**) A representative side view AFM image of IL film fabricated by IBFW. The inset blue square illustrates the entrance region of liquid reservoir and the liquid film. (**c**) The curvature of liquid-gas interface of the blue box part in (b), the maximum of curvature occurs at the film entrance and the capillary force can be calculated applying Young-Laplace equation. (**d**) The plots of disjoining pressure from different contributions. The blue hollow triangles depict the Van der Walls contribution, or in other words, the nonpolar effect; the yellow squares with cross lines depict the acid component of disjoining pressure i.e. the polar contribution; the green hollow circles represent the Born repulsion contribution which only dominates at extremely thin film thickness. The red triangles represent the summation of the aforementioned components. The yellow rectangle regions indicate the range of capillary force calculated by curvature values of different liquid film profiles and the consequent film thickness range falls into 10 nm ~ 30 nm with 20 nm the most common results which agrees with our experimental observations.

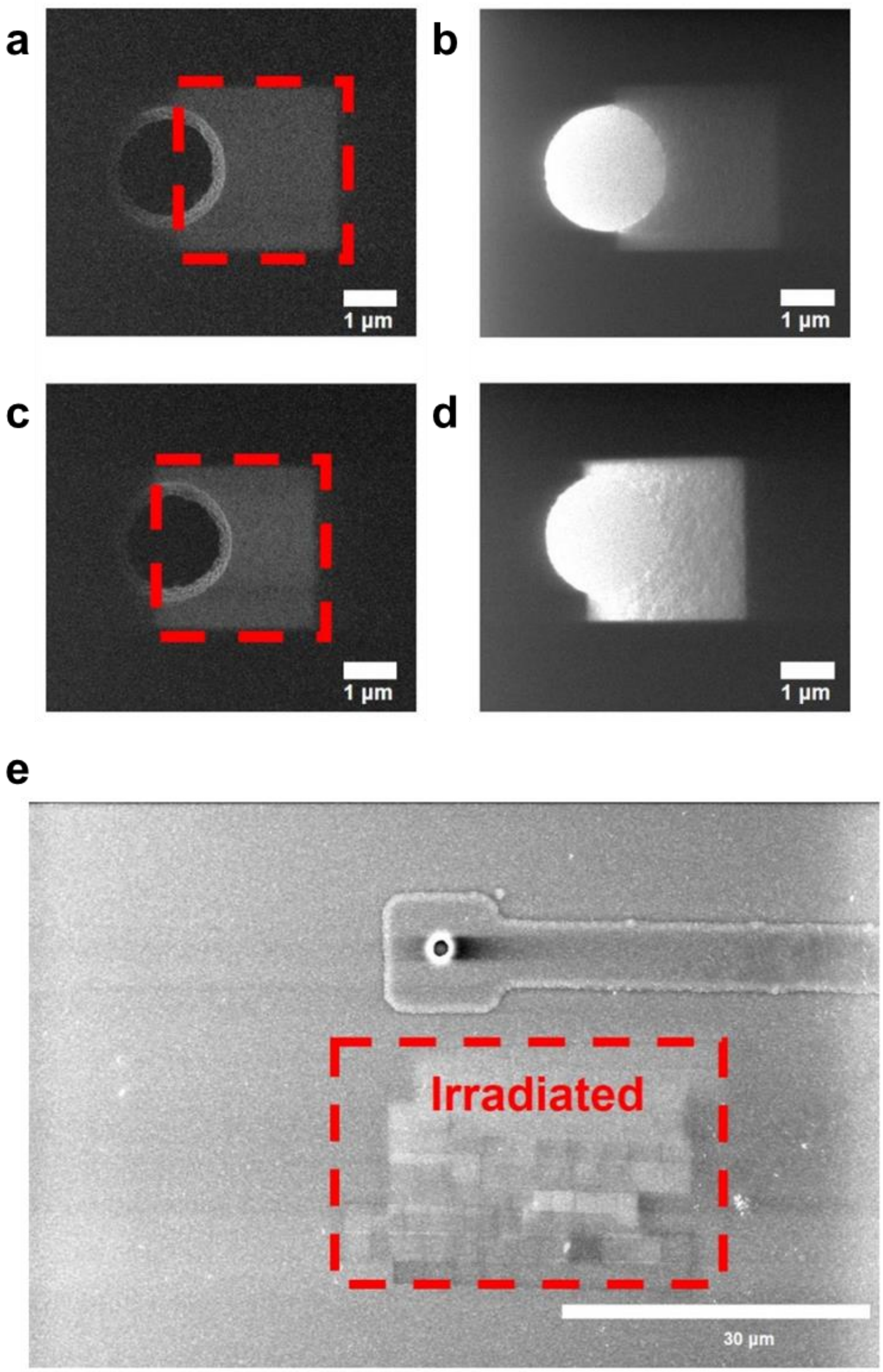

Supplementary Figure 16: Comparison between IBFW and surface wettability modification. (**a**) HIM image of micro hole and 16.8 $pC/\mu m^2$ HFIB irradiated area; (**b**) HIM image of the identical substrate with the liquid reservoir and liquid channel filled with [EMIM][DCA]; (**c, d**) are similar but with the red square area irradiated by 84 $pC/\mu m^2$ HFIB; (**e**) ESEM image of an example of the morphology of a same substrate irradiated by 84 $pC/\mu m^2$ HFIB.

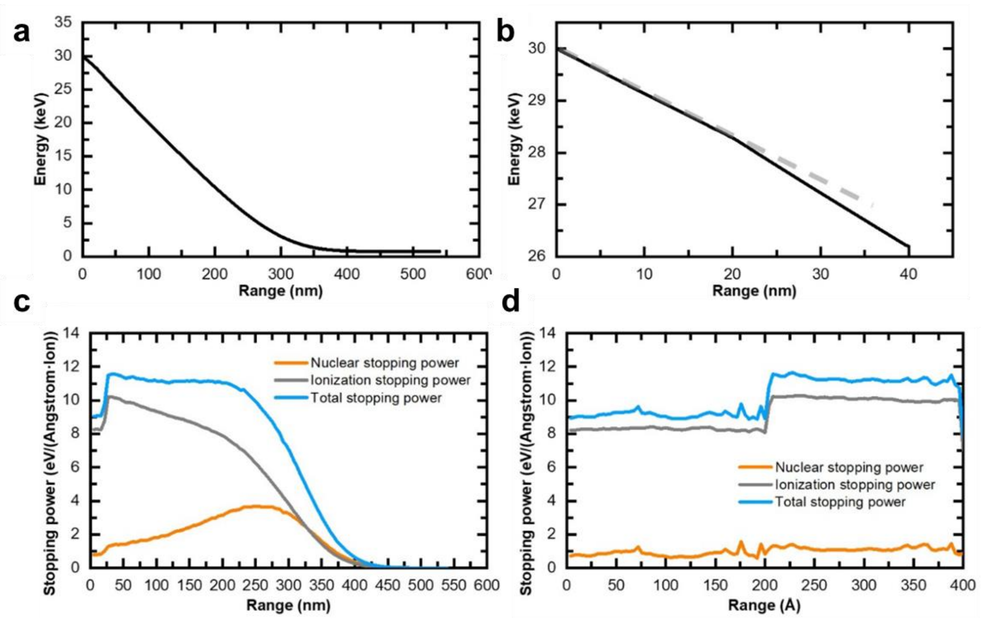

Supplementary Figure 17: Monte Carlo simulation results employing SRIM software.

(**a**) He energy vs. depth into IL-SiO_2 system; (**b**) the energy loss near the silicon dioxide surface; (**c**) the energy loss of He ions vs. depth; (**d**) the energy loss near IL-SiO_2 interface.

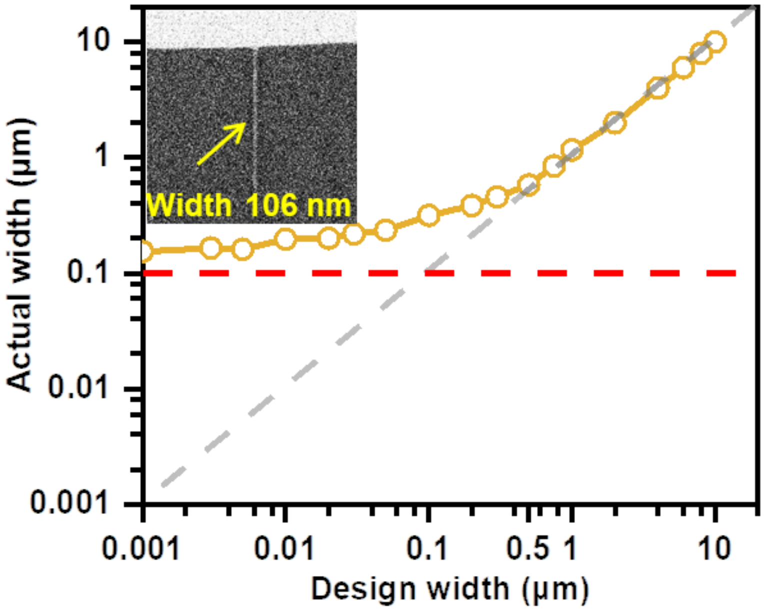

Supplementary Figure 18: The minimal width limitation of the IBFW liquid film.

The linewidth limitation reaches 100 nm and is represented by the red dash line. The inset shows an example of the narrowest liquid film fabricated by IBFW with a line width of 106 nm, and length of 10 μm. For design widths above 500 nm, the actual film width matches the design width. However, when the design width reaches 500 nm, the actual film width broadens by about 10%. The film width deviates more from the design width as the design width decreases, until it reaches its minimal limitation.

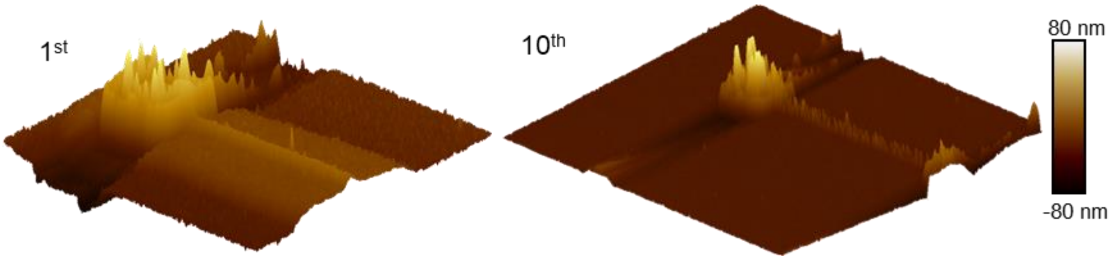

Supplementary Figure 19: AFM images of the IBFW liquid film from rewritable tests.

The AFM tapping mode images (10 μm × 10 μm) of the IBFW film from the 1st and the 10th rewritable tests, with the color scale indicates the height.

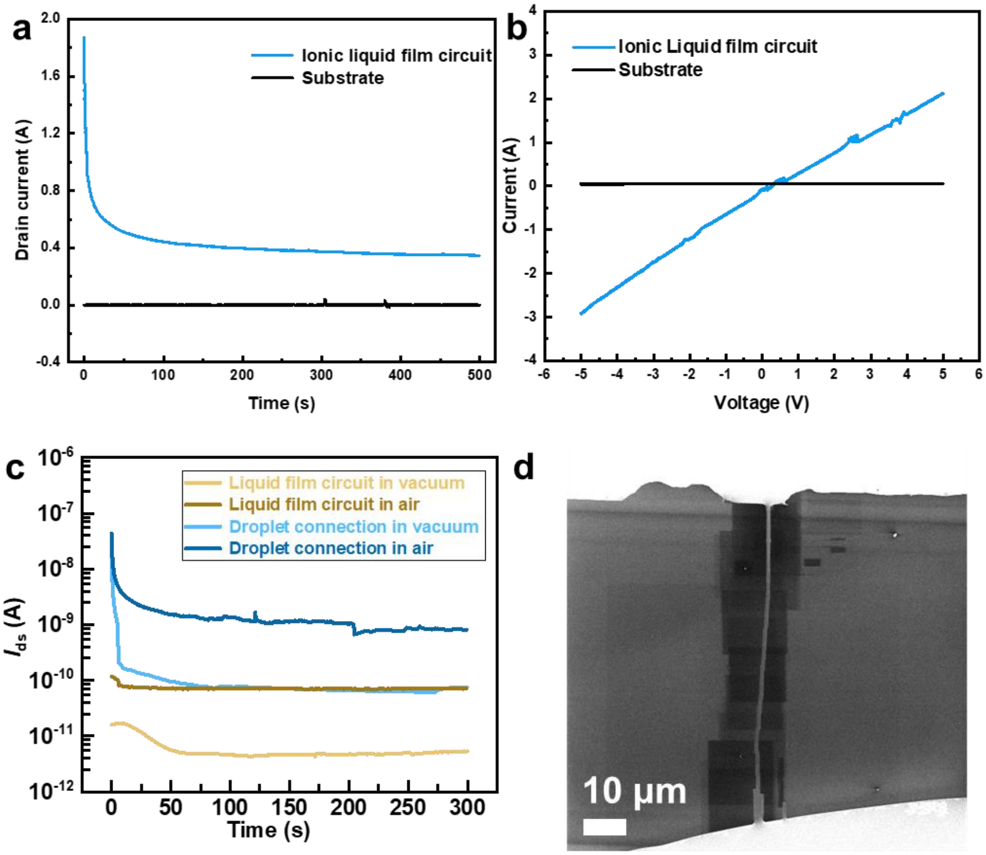

Supplementary Figure 20: Electrochemistry characteristics analysis of IL nano film circuit. **(a)** drain current between Au electrodes vs. time, the black line represents an identical measurement with no liquid film connect the electrodes; **(b)** Linear sweep volt-ampere curve of liquid film circuit; **(c)** The transient current curves of IL film circuit and droplet circuit in high vacuum and in atmospheric environment as a function of time, the source-drain voltage is 10 V. Both the droplet circuit and nanofilm circuit have a higher current in atmosphere than in vacuum chamber, this is due to the water molecules adsorbed by the droplet and the film that react at the electrode surface and create a reaction current. The result in vacuum chamber is more intriguing. The adsorption-reaction current has been disentangled, and the current we measured is solely contributed by the formation of electric double layer. As shown by the yellow curve, the EDL formation current curve of nanofilm circuit quickly decreases to be indiscernible, which indicate that the EDL in nanofilm circuit established quickly and reaches an equilibrium. On the contrary, the EDL formation current of droplet circuit decrease much slower, and keep on decreasing at the end of the 300 s measurement, which indicate that the equilibrium is hard to attain in a droplet circuit. Such differences explain that nano film circuit with a much shorter relaxation time is more suitable for gas sensing purpose. **(d)** The HIM image of the liquid nano circuit employed in this experiment.

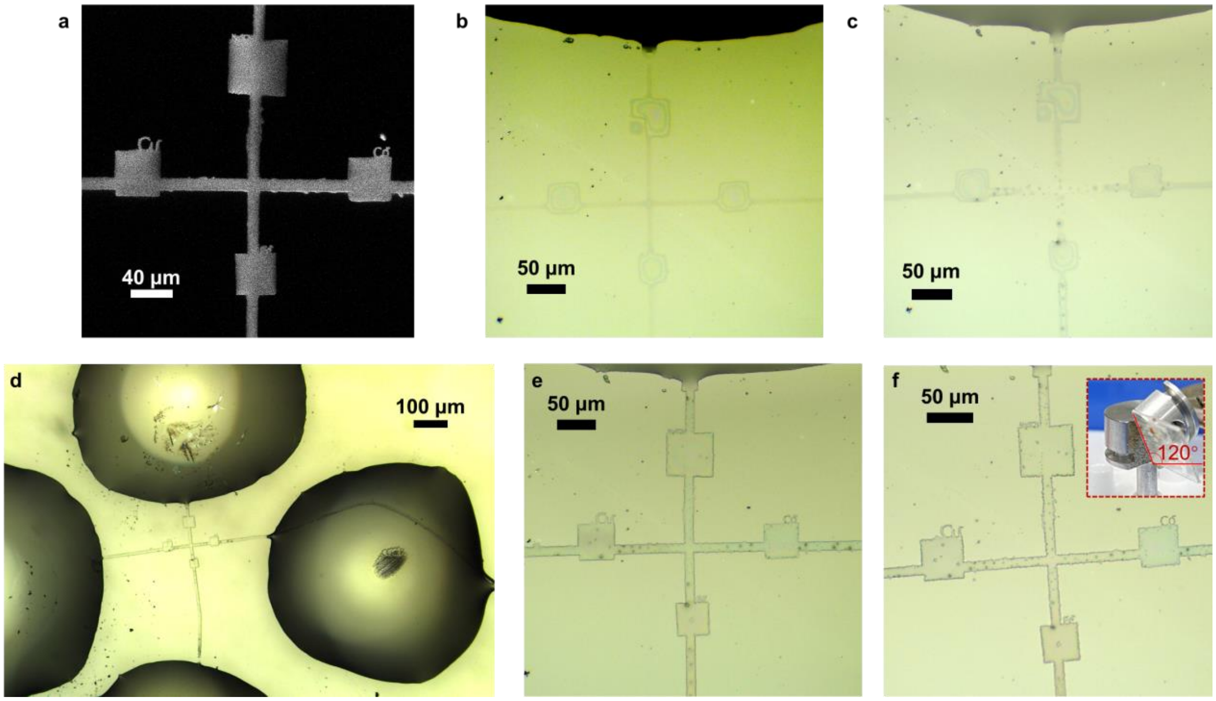

Supplementary Figure 21: Time series images of in-situ chemical reaction chip.

(a) HIM image of channels right after the IBFW fabrication. **(b)** The fluid channel first transferred to the optical microscope; the colorless transparent fluid channel is a bit hard to be distinguished from the transparent silica substrate. **(c)** After the injection of all solutions and reacts in atmosphere for 5 min. **(d)** The whole picture of the reaction chip before transferred to vacuum chamber. **(e)** 48 h after metal ions injection and storing in vacuum. After stored in vacuum chamber, the water has been eliminated, and the color of the different complexes become obvious. The sample goes through several times of transferring between vacuum chamber and air environment and the injection of analyte solutions into droplet reservoirs, while the liquid film pattern remains barely changed through the experiments. Such results demonstrate the stability of IBFW liquid film channel to the exposure of air environment and to non-direct physical contact for solution injections. **(f)** The reaction chip one month after injection (with one week for gravity stability test). The chip is mounted on a customized sample holder which can adjust the tilting angle from 0-180° to test the IBFW film stability against gravity. The inset shows the mounted sample with tilting angle 120° and is stored for one week.

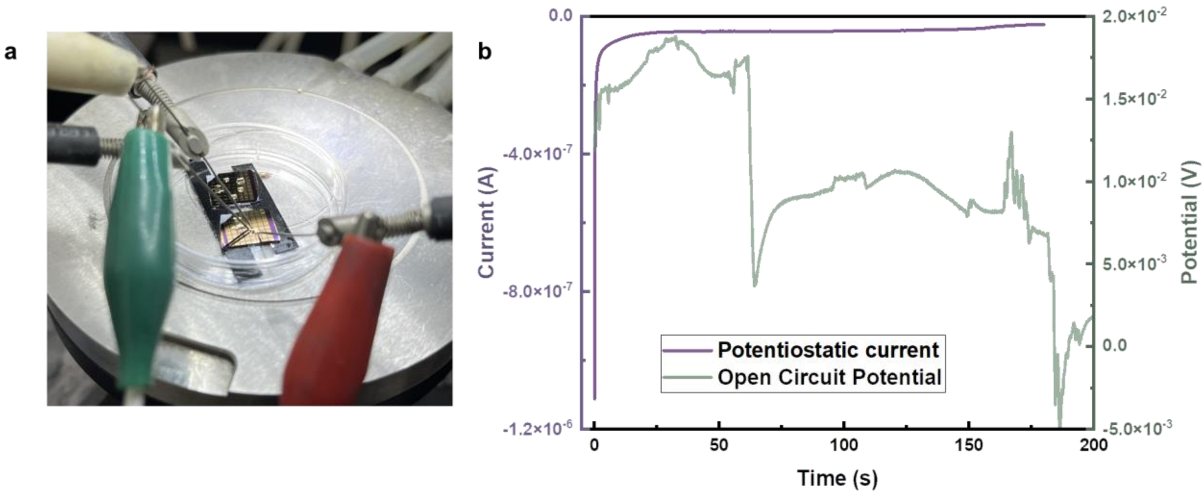

Supplementary Figure 22: The electrodeposition experiments of AgTCNQ.
(a) The three-electrode configuration adopted in current work. **(b)** The green line is the V-t curve of AgTCNQ open circuit potential measurement, which serves as a reference for the subsequent experiments. The purple line is the deposition current vs. time curve of AgTCNQ deposition at constant potential, -0.1 V (vs. Ag).

Supplementary Table 1.

The ion beam scan parameters in IBFW experiment.

The HIM can adjust its HFIB dose density in a broad range. Important parameters include: beam current, I; dwell time, τ, that the beam remains at each dwell point before moving to the next; pixel spacing, s, the physical spacing between two contiguous dwell points. The following schematic of a standard scanning array illustrates the horizontal spacing, s_H, the vertical spacing, s_V, and the scan sequence of series of scan points. Three other scan modes are also illustrated in the following figures.

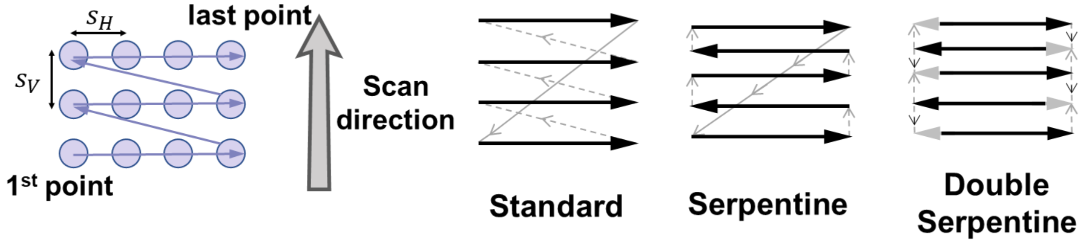

The optimized ion beam parameters

Beam parameters	value
Accelerating voltage	$30\ kV$
Beam current	$0.1 - 1\ pA$
Dwell time	$0.5 - 2\ \mu s$
Flood gun	Open
Pixel size	1024×1024
Scan direction	Left-to-right in row, then row by row
Scan mode	Point by point

Supplementary Table 2.
The physicochemical parameters of [EMIM][DCA]

Physical property	Value	Note
Melting point	-21 ℃	
density	$1.11\ g/cm^3$	
Surface tension	$47.3\ mN/m$	20 ℃
Decomposition temperature	104 ℃	
Electrical conductivity	$28\ mS/cm$	25 ℃
Viscosity	$21\ cP$	20 ℃
Polarity	$51.7\ kcal/mol$	
Electrochemical window	$3.3\ V$	
Refractive index	1.5307	

Supplementary Table 3.

Parameters of 4 RTILs employed (20 ℃)

Name	MW (g/mol)	Surface Tension (mN/m)	Density (g/cm^3)	Viscosity (cP)
[EMIM][DCA]	177.21	47.3	1.11	21
[EMIM]BF_4	197.97	49	1.294	45
[BMIM][PF_6]	284.18	38	1.37	284.18
[EMIM][NTf_2]	391.31	36	1.53	32

Supplementary Table 4.
Wetting status of different RTILs on three solid surfaces (contact angle in degree, 20 °C)

Name	PECVD SiO_2	TOX SiO_2	10 nm Au 5nm Ti on SiO_2
[EMIM][DCA]	40 ± 4	71 ± 6.4	47 ± 4.5
$[EMIM]BF_4$	36 ± 4.8	58 ± 3.4	55 ± 4.1
$[BMIM]PF_6$	83 ± 6.3	63 ± 2.1	68 ± 8.9
$[EMIM][NTf_2]$	36 ± 3.9	59 ± 5.7	38 ± 4.1

References

1. Chen, Y., Zhao, Z. & Liu, Y. Wettability characteristic of PTFE and glass surface irradiated by keV ions. *Appl. Surf. Sci.* **254**, 5497–5500 (2008).
2. Fox, D. *et al.* Helium ion microscopy of graphene: beam damage, image quality and edge contrast. *Nanotechnology* **24**, 335702 (2013).
3. Penkov, O. V. *et al.* Ion-beam irradiation of DLC-based nanocomposite: Creation of a highly biocompatible surface. *Appl. Surf. Sci.* **469**, 896–903 (2019).
4. Inoue, Y., Yoshimura, Y., Ikeda, Y. & Kohno, A. Ultra-hydrophobic fluorine polymer by Ar-ion bombardment. *Colloids Surf. B Biointerfaces* **19**, 257–261 (2000).
5. Li, Y. *et al.* Ultrafast Diameter-Dependent Water Evaporation from Nanopores. *ACS Nano* **13**, 3363–3372 (2019).
6. Cazabat, A. M., Heslot, F., Troian, S. M. & Carles, P. Fingering instability of thin spreading films driven by temperature gradients. *Nature* **346**, 824–826 (1990).
7. Liu, G. L., Kim, J., Lu, Y. & Lee, L. P. Optofluidic control using photothermal nanoparticles. *Nat. Mater.* **5**, 27–32 (2006).

8. Ziegler, J. F., Ziegler, M. D. & Biersack, J. P. SRIM – The stopping and range of ions in matter (2010). *Nucl. Instrum. Methods Phys. Res. Sect. B Beam Interact. Mater. At.* **268**, 1818–1823 (2010).
9. Klomfar, J., Součková, M. & Pátek, J. Temperature Dependence of the Surface Tension and Density at 0.1 MPa for 1-Ethyl- and 1-Butyl-3-methylimidazolium Dicyanamide. *J. Chem. Eng. Data* **56**, 3454–3462 (2011).
10. Yogev, S. *et al.* Charging of dielectrics under focused ion beam irradiation. *J. Appl. Phys.* **103**, 064107 (2008).
11. Coffman, C. S., Martínez-Sánchez, M. & Lozano, P. C. Electrohydrodynamics of an ionic liquid meniscus during evaporation of ions in a regime of high electric field. *Phys. Rev. E* **99**, 063108 (2019).
12. Zhang, F. *et al.* Electric-Field-Driven Ion Emission from the Free Surface of Room Temperature Ionic Liquids. *J. Phys. Chem. Lett.* **12**, 711–716 (2021).
13. Magnani, M. & Gamero-Castaño, M. Energy barrier for ion field emission from a dielectric liquid sphere. *Phys. Rev. E* **105**, 054802 (2022).
14. Greenwood, G. *et al.* Effects of Layering and Supporting Substrate on Liquid Slip at the Single-Layer Graphene Interface. *ACS Nano* **15**, 10095–10106 (2021).
15. An, R. *et al.* Atomic force microscopy probing interactions and microstructures of ionic liquids at solid surfaces. *Nanoscale* **14**, 11098–11128 (2022).
16. He, Y., Li, H., Qu, C., Cao, W. & Ma, M. Recent understanding of solid-liquid friction in ionic liquids. *Green Chem. Eng.* **2**, 145–157 (2021).

17. Di Lecce, S., Kornyshev, A. A., Urbakh, M. & Bresme, F. Electrotunable Lubrication with Ionic Liquids: the Effects of Cation Chain Length and Substrate Polarity. *ACS Appl. Mater. Interfaces* **12**, 4105–4113 (2020).

18. Bhatt, B., Gupta, S., Sumathi, V., Chandran, S. & Khare, K. Electric Field Driven Reversible Spinodal Dewetting of Thin Liquid Films on Slippery Surfaces. *Adv. Mater. Interfaces* 2202063 (2023).

19. London, F. Zur Theorie und Systematik der Molekularkräfte. *Z. Für Phys.* **63**, 245–279 (1930).

20. Tanabe, I., Kurawaki, Y., Morisawa, Y. & Ozaki, Y. Electronic absorption spectra of imidazolium-based ionic liquids studied by far-ultraviolet spectroscopy and quantum chemical calculations. *Phys. Chem. Chem. Phys.* **18**, 22526–22530 (2016).

21. Paschoal, V. H., Faria, L. F. O. & Ribeiro, M. C. C. Vibrational Spectroscopy of Ionic Liquids. *Chem. Rev.* **117**, 7053–7112 (2017).

22. Hunger, J., Stoppa, A., Schrödle, S., Hefter, G. & Buchner, R. Temperature Dependence of the Dielectric Properties and Dynamics of Ionic Liquids. *ChemPhysChem* **10**, 723–733 (2009).

23. Canongia Lopes, J. N., Deschamps, J. & Pádua, A. A. H. Modeling Ionic Liquids Using a Systematic All-Atom Force Field. *J. Phys. Chem. B* **108**, 2038–2047 (2004).

24. Canongia Lopes, J. N. & Pádua, A. A. H. Molecular Force Field for Ionic Liquids Composed of Triflate or Bistriflylimide Anions. *J. Phys. Chem. B* **108**, 16893–16898 (2004).

25. Canongia Lopes, J. N. & Pádua, A. A. H. Molecular Force Field for Ionic Liquids III: Imidazolium, Pyridinium, and Phosphonium Cations; Chloride, Bromide, and Dicyanamide Anions. *J. Phys. Chem. B* **110**, 19586–19592 (2006).

26. Canongia Lopes, J. N., Pádua, A. A. H. & Shimizu, K. Molecular Force Field for Ionic Liquids IV: Trialkylimidazolium and Alkoxycarbonyl-Imidazolium Cations; Alkylsulfonate and Alkylsulfate Anions. *J. Phys. Chem. B* **112**, 5039–5046 (2008).

27. Martínez, L., Andrade, R., Birgin, E. G. & Martínez, J. M. PACKMOL: A package for building initial configurations for molecular dynamics simulations. *J. Comput. Chem.* **30**, 2157–2164 (2009).

28. Evans, D. J. & Holian, B. L. The Nose–Hoover thermostat. *J. Chem. Phys.* **83**, 4069–4074 (1985).

29. Stukowski, A. Visualization and analysis of atomistic simulation data with OVITO–the Open Visualization Tool. *Model. Simul. Mater. Sci. Eng.* **18**, 015012 (2009).

30. Allen, D. *et al.* An investigation of the radiochemical stability of ionic liquids. *Green Chem.* **4**, 152–158 (2002).

31. Xue, Z., Qin, L., Jiang, J., Mu, T. & Gao, G. Thermal, electrochemical and radiolytic stabilities of ionic liquids. *Phys. Chem. Chem. Phys.* **20**, 8382–8402 (2018).
