# Supplementary material for: Rewritable printing of ionic liquid nanofilm utilizing focused ion beam induced film wetting
Source: Nat Commun. 2024 Apr 5;15:2949. doi: 10.1038/s41467-024-47018-9 (PMC10997651; doi:10.1038/s41467-024-47018-9)
Supplement: Supplementary file 4 — Description of Additional Supplementary Files [file 41467_2024_47018_MOESM4_ESM.pdf]

Description of Additional Supplementary Files

Supplementary Movie Legends:

Supplementary Movie 1. Time series of film propagation process after scanned with standard scan array. The ion beam moves parallel to the contact line of ionic liquid droplet reservoir, then move vertically and return to the horizontal starting position to scan the next line of scan spots. Such process is repeated until the whole designed area has been scanned. The branches of IL tilt to the left-bottom direction, then the rivulets broaden and finally irrigate the entire scanned area. A uniform liquid film with desired rectangular pattern can be seen in the last frame.

Supplementary Movie 2. Time series of film propagation process after scanned with serpentine scan array. The ion beam moves parallel to the contact line of ionic liquid droplet reservoir, then move vertically to scan the next line of scan spots but in reverse direction. The branches flow characters and process are similar to the situation with standard scan array, expect that the branches' lateral tilting angle is relatively less obvious.

Supplementary Movie 3. Time series of film propagation process after scanned with unsuccessful scan array. Unlike the situations in Supplementary Movie 1,2, where the ion beam first moves parallel to the contact line of droplet reservoir, when the ion beam first moves perpendicular to then parallel to the CTL, the desired liquid film pattern cannot be induced, as can be seen in the last frame and Supplementary Fig. 5 c,d. As an example, the ion beam first moves vertically to CTL and towards the droplet, then move to the next column of scan spot and return to the starting vertical position and repeat such process. The left-top area of designed scan area is not filled with ionic liquid as can be seen in the last frame.

Supplementary Movie 4. Schematics of the tilting of branches flow and scan area that cannot be filled. When the speed of ion beam is much faster compared with the film propagation, the vertical scan direction can guarantee the liquid film can follow the external field stimulus. Consequently, the liquid film can only propagate small distances at the first several columns of scan spots. But when the ion beam return to the starting vertical scan position and move outwards, the previously propagated liquid film would first be attracted backwards then be induced further into the desired direction. The consequent branches of liquid flow tilt to the left-bottom direction and small branches tilt backwards to the contact line, as can be seen in Supplementary Fig. 5 d. The analysis can be adapted to explain other branches flow tilting directions and liquid filled area.
